# Verification of EZH2 as a druggable target in metastatic uveal melanoma

**DOI:** 10.1186/s12943-020-01173-x

**Published:** 2020-03-04

**Authors:** Bei Jin, Ping Zhang, Hailin Zou, Huijing Ye, Yun Wang, Jing Zhang, Huasheng Yang, Jingxuan Pan

**Affiliations:** State Key Laboratory of Ophthalmology, Guangdong Provincial Key Laboratory of Ophthalmology and Visual Science, Zhongshan Ophthalmic Center, Sun Yat-sen University, 54 South Xianlie Road, Guangzhou, 510060 People’s Republic of China

**Keywords:** Uveal melanoma, EZH2, Hepatic metastasis, Motility, Cancer stem-like cells

## Abstract

**Background:**

Hepatic metastasis develops in ~ 50% of uveal melanoma (UM) patients with no effective treatments. Although GNAQ/GNA11 mutations are believed to confer pathogenesis of UM, the underlying mechanism of liver metastasis remains poorly understood. Given that profound epigenetic evolution may occur in the long journey of circulating tumor cells (CTCs) to distant organs, we hypothesized that EZH2 endowed tumor cells with enhanced malignant features (e.g., stemness and motility) during hepatic metastasis in UM. We aimed to test this hypothesis and explore whether EZH2 was a therapeutic target for hepatic metastatic UM patients.

**Methods:**

Expression of EZH2 in UM was detected by qRT-PCR, Western blotting and immunohistochemistry staining. Proliferation, apoptosis, cancer stem-like cells (CSCs) properties, migration and invasion were evaluated under circumstances of treatment with either EZH2 shRNA or EZH2 inhibitor GSK126. Antitumor activity and frequency of CSCs were determined by xenografted and PDX models with NOD/SCID mice. Hepatic metastasis was evaluated with NOG mice.

**Results:**

We found that EZH2 overexpressed in UM promoted the growth of UM; EZH2 increased the percentage and self-renewal of CSCs by miR-29c-DVL2-β-catenin signaling; EZH2 facilitates migration and invasion of UM cells via RhoGDIγ-Rac1 axis. Targeting EZH2 either by genetics or small molecule inhibitor GSK126 decreased CSCs and motility and abrogated the liver metastasis of UM.

**Conclusions:**

These findings validate EZH2 as a druggable target in metastatic UM patients, and may shed light on the understanding and interfering the complicated metastatic process.

## Introduction

Metastasis is a non-evadable and tragic ending in most patients with solid tumors. The mortal complex process of metastasis starting from primary tumor foci to clinically overt metastatic foci at least includes intricate steps such as intravasation, circulation, extravasation, and colonization in distant organs [1]. CTCs in bloodstream gaining stemness and resistance to apoptosis may enter distant organ tissues to become metastasis initiating cells (MICs) to colonize. In clinic, the specific single organ tropism metastasis (e.g., uveal melanoma and pancreatic ductal adenocarcinoma to liver) may offer us a simplified model for understanding and interfering the extraordinarily complicated metastatic process.

Single organ liver metastasis in ~ 85% metastatic patients with UM is a prominent clinical characteristic and thereby a natural model. UM originated from melanocytes of the choroid, ciliary and iris, is the most common primary ocular malignancy in adults [2]. Despite effective regimens for primary tumor, the 5-year survival rate of UM remained almost unchanged for the past three decades [3]. Mutually exclusive gain-of-function mutations in GNAQ/GNA11 found in 80% of UM, which activate multiple pathways, resulting in activation of MAPK and YAP to promote the growth of UM [4], presumably drive the pathogenesis of UM. Genetic analysis revealed that inactivating somatic mutation in BAP1 is a significant predictor for metastatic UM [5]. Neddylation modification is reported to be involved in the hepatic metastasis in UM and is a promising therapeutic target [6]. It remains, however, largely enigmatic about the underlying mechanism and practical targeting approaches of the liver metastasis in UM.

Enhancer of zeste homolog 2 (EZH2), an essential component in epigenetic polycomb repressive complex 2 (PRC2), is required for maintaining CSC properties showing enrichment of CSC, enhanced self-renewal ability and particular transcriptional pattern in multiple types of cancers including breast cancer [7], glioblastoma [8] and pancreatic ductal adenocarcinoma [9]. EZH2 is also essential for acquisition of cell motility and positively regulates genes enriched for cytoskeletal components counting for invasive cell population and promotes cutaneous melanoma motility and metastasis [10].

Elevated levels of EZH2 predict dismal prognosis with high risk of metastasis and shorter survival in a cohort of 89 UM patient samples [11]. Given the potency of EZH2 in strengthening stemness of CSCs and motility, we, in the present study, hypothesized that EZH2 endowed tumor cells with enhanced malignant features during liver metastasis in UM. We found that EZH2 overexpressed in UM promoted the growth of UM; increased the percentage and self-renewal of CSCs by miR-29c-DVL2-β-catenin signaling; facilitated migration and invasion via RhoGDIγ-Rac1 axis. Targeting EZH2 either by genetics or small molecule inhibitor GSK126 decreased CSCs and motility and abrogated the liver metastasis of UM. These findings validate EZH2 as an attractive therapeutic target against hepatic metastasis of UM.

## Materials and methods

### Cell culture

UM cell lines 92.1, Mel270, Omm1 and Omm2.3 were kindly provided by Dr. M. J Jager, Leiden University Medical Center, Leiden, The Netherlands. MP41 cells were from ATCC (Manassas, VA). UM cell lines were cultured in RPMI 1640 medium (Invitrogen, Shanghai, China) supplemented with 10% fetal bovine serum (FBS, Hyclone, Guangzhou, China), 2 mmol/L L-glutamine, 100 units/ml penicillin, and 100 μg/ml streptomycin [3, 12]. 293 T cells were cultured in DMEM supplemented with 10% FBS. Cells were kept at 37 °C in a humidified incubator with 5% CO_2_. All the cell lines were tested and authenticated by short tandem repeat (STR) matching analysis of cells. No cross-contamination of other human cells was found in these cells. No mycoplasma (Thermo-Fisher Scientific) contamination was detected.

### Patient samples

UM patient specimens for immunohistochemical (IHC) staining were collected from 2002 to 2005 in Zhongshan Ophthalmic Center of Sun Yat-sen University, after informed written consent was obtained from each subject or each subject’s guardian according to the institutional guidelines and the Declaration of Helsinki principles. Tumor tissue sections were stained with anti-EZH2 or anti-HMB45 antibody for IHC analysis. The association between EZH2 expression and clinicopathologic features in UM patients was summarized in Supplementary Table S1.

For Western blotting analysis of EZH2 in UM, malignant uveal tissue from enucleation eyes of UM patient or normal uveal tissues of healthy donors were prepared in RIPA buffer [13].

### Uveal melanoma PDX

MP41 cells (1 × 10^7^ cells/mouse) were subcutaneously inoculated in the flank of NOD/SCID mice to allow formation of the primary tumors. When reached ~ 1000 mm^3^, xenografted tumors were removed and cut into ~ 30 mm^3^ slices and consecutively implanted into the secondary recipient NOD/SCID mice for rounds of mouse-to-mouse passage [14]. The tertiary recipient NOD/SCID mice bearing MP41 PDX were randomly grouped (*n* = 8/group) for either placebo (20% captisol, i.p., 4 w) or GSK126 (50 mg/kg/day, i.p., 4 w). Tumor growth was monitored every 2 days.

### Statistical analysis

GraphPad Prism 5.0 (San Diego, CA) was used for statistical analysis. All experiments were performed at least three times, and results were expressed as mean ± standard error of the mean (SEM) unless otherwise stated. Comparison between 2 groups was analyzed by Student’s *t* test and between more than 2 groups by one-way ANOVA with *post hoc* comparison by Tukey test, respectively. *P* < 0.05 was considered statistically significant.

## Results

### EZH2 is overexpressed in UM and promotes proliferation of UM

**Fig. 1:**
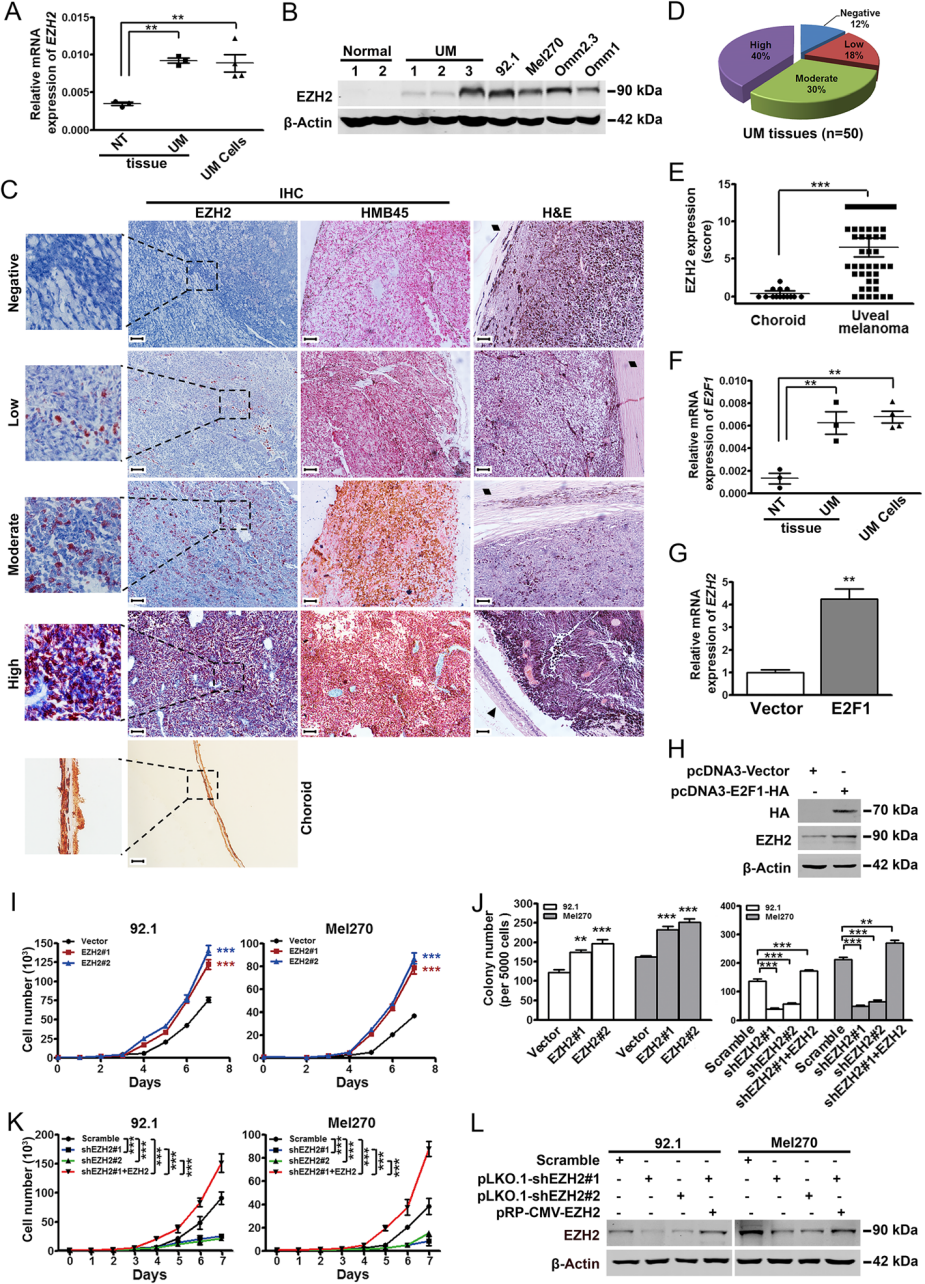
EZH2 is overexpressed in UM. **a-b** The mRNA (**a**) and protein (**b**) levels of EZH2 in normal choroid of healthy donors (3 for qRT-PCR, 2 for Western blotting analysis), UM tissues (*n* = 3) and UM cells (92.1, Mel270, Omm2.3 and Omm1). Each dot represents one sample or one cell line. Data are mean ± SEM. **, *P* < 0.01, one-way ANOVA, *post hoc* intergroup comparisons. **c-e** IHC staining of EZH2 in paraffin embedded UM tissues. Choroid was adjacent normal tissue. H&E staining and IHC analysis of HMB45 were the same samples as those for EZH2 staining. ♦, sclera; ▲, retina. Scale bar, 100 μm, Olympus IX71 (**c**). The percentage of UM patients was divided by scores of EZH2 expression (**d**). Data are mean ± SEM. ***, *P* < 0.0001, Student’s *t* test (**e**). **f-h** E2F1 conferred EZH2 overexpression in UM. E2F1 expression was detected by qRT-PCR in normal choroid of healthy donors (*n* = 3), UM tissues (*n* = 3) and UM cells (*n* = 4). Each dot represents one sample or one cell line. Data are mean ± SEM. **, *P* < 0.01, one-way ANOVA, *post hoc* intergroup comparisons (**f**). Ectopic expression of E2F1 in Mel270 led to aberrant expression of EZH2 as detected by qRT-PCR (**g**) and Western blotting analysis (**h**). Data are mean ± SEM. **, *P* < 0.01, Student’s *t* test. **i-l** EZH2 promoted proliferation of UM cells. UM cells transfected with vector, EZH2-encoding constructs (**i**), scramble or lentiviral shRNAs against EZH2 with or without EZH2 restored (**j-l**) were subjected to either cellular growth determination (**i** and **k**), colony formation evaluation (**j**) or Western blotting analysis (**l**). Data are mean ± SEM. **, *P* < 0.01; ***, *P* < 0.0001, one-way ANOVA, *post hoc* intergroup comparisons

We collected choroidal tissues from an eye of healthy donors and cancerous tissues of patients with UM. A prominently elevated expression of EZH2 in the UM cells and tissues versus the normal counterparts was observed (Fig. 1a-b). The *in-situ* expression status of EZH2 in the primary specimens were detected by IHC staining. The positive staining of EZH2 was observed in 44 out of 50 (88%) of the tested UM cases which were melanocyte-specific HMB45-positive staining (Fig. 1c-d). The expression of EZH2 in the UM tissues were significantly elevated comparing with that in adjacent normal choroid (Fig. 1e). EZH2 were positively correlated with the largest basal diameter and thickness of primary tumors (*P* < 0.05, Supplementary Table S1), which are predictors of increasing risk of metastasis [15]. Analysis of UM in TCGA dataset (*n* = 77) revealed a significantly reduced overall survival in the EZH2-overexpressed cases (Supplementary Fig. S1A). These results reveal that the elevated EZH2 in UM predicts a worse prognosis.

EZH2 is a target gene of transcriptional factor E2F1 in a set of human tumors [16]. We asked whether E2F1 conferred EZH2 overexpression in UM cells. E2F1 was prominently overexpressed in UM tissues and cells comparing to that in normal choroid (Fig. 1f). Ectopic expression of E2F1 resulted in a significantly increase in mRNA (Fig. 1g) and protein levels (Fig. 1h) of *EZH2*. These data suggest that E2F1 impels EZH2 expression in UM.

We next examined the functional role of EZH2 in UM. The levels of EZH2 and H3K27me3 were enhanced in EZH2-transfected UM cells (Supplementary Fig. S2A). UM cells overexpressing EZH2 displayed an escalated cellular proliferation trait than those with empty vector as reflected by the increased cell numbers (Fig. 1i) and clonogenecity (Fig. 1j, *left*). In contrast, EZH2 knockdown decreased the levels of H3K27me3 (Supplementary Fig. S2B). Correspondingly, the clonogenicity (Fig. 1j, *right*) and growth (Fig. 1k) were impeded in the EZH2-silenced cells. Cell growth and clonogenicity were restored in shEZH2#1-bearing cells when EZH2 expression was rescued (Fig. 1j-l). These results indicate that EZH2 promotes proliferation of UM cells, and that EZH2 is a potential therapeutic target.

### Pharmacologic inhibition of EZH2 suppresses proliferation of UM cells

**Fig. 2:**
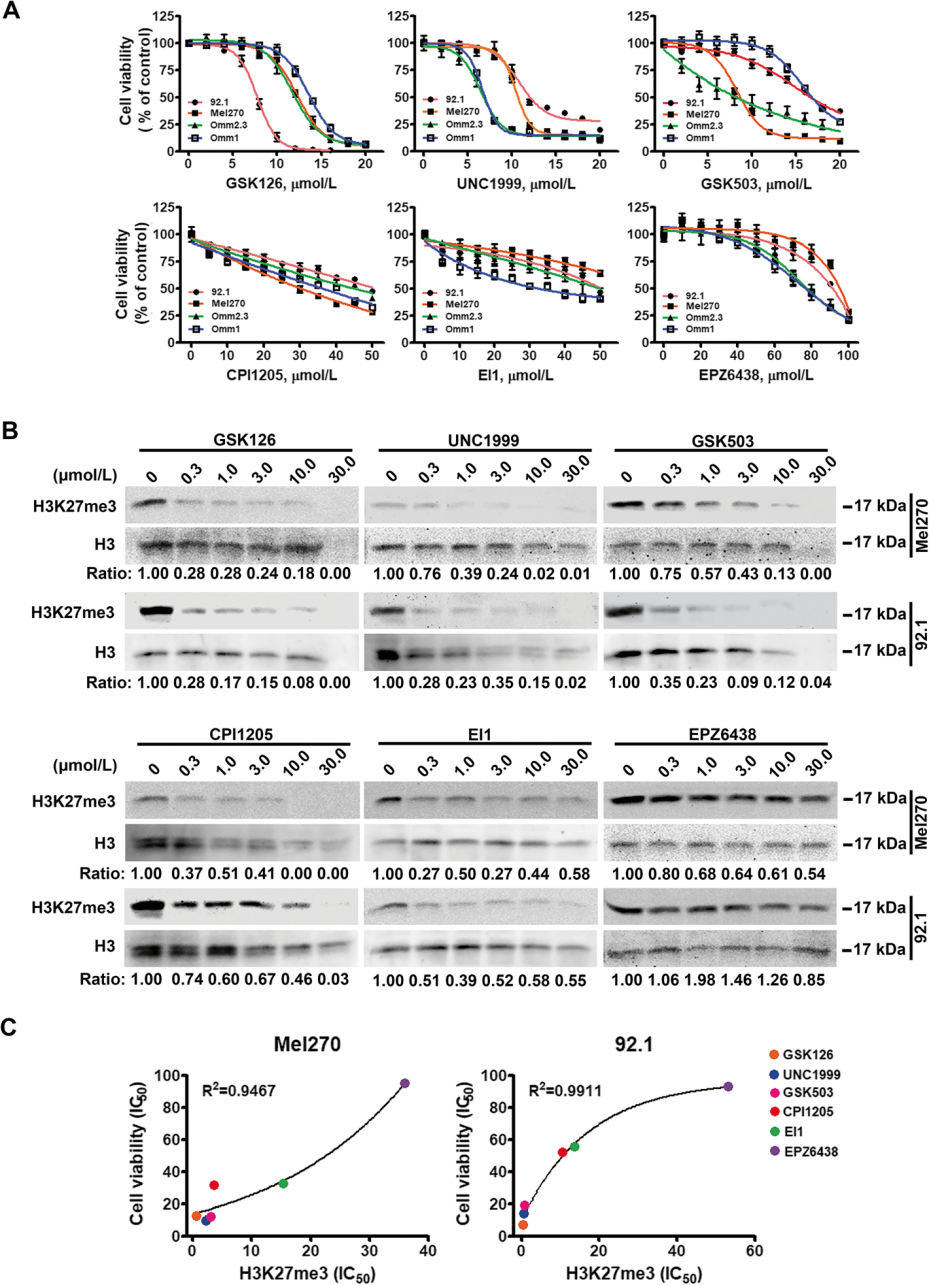
Pharmacologic inhibition of EZH2 prohibits growth of UM cells**. a** Targeting EZH2 by HMT inhibitors suppressed proliferation. UM cells were exposed to various concentrations of inhibitors for 72 h. Cell viability was determined by MTS assay. **b-c** HMT inhibitors eliminated H3K27me3 in UM. UM cells were treated with HMT inhibitors (μmol/L) as indicated for 72 h, and then subjected to Western blotting analysis. The expression levels of H3K27me3 in HMT inhibitor-treated cells comparing to those of control ones were analyzed by Image J (**b**). Plots were analyzed by IC_50_ values of HMT inhibitors in downregulating H3K27me3 expression against those in inhibiting cell viabilities of UM cells. The dots along X and Y axis represented treatment with GSK126, UNC1999, GSK503, CPI1205, EI1 and EPZ6438, respectively (**c**)

To investigate whether EZH2 was a potential target, we examined a series of histone methyltransferase (HMT) inhibitors on UM cells. The response of UM cells to these HMT inhibitors varies in terms of the growth curves (Fig. 2a) and IC_50_ values (Supplementary Table S2). H3K27me3 expression was significantly declined with treatment of HMT inhibitors, which was intensively correlated with the effect of growth suppression (Fig. 2b-c, R^2^ > 0.9). These results support that EZH2 may be a therapeutic target in UM.

Given that GSK126 is one of the three HMT inhibitors in clinical trials (http://clinicaltrials.gov), we next assessed the inhibitory impact of GSK126 on the anchorage-independent growth and cell cycle distribution of UM cells. GSK126 caused to reduced clonogenecity (Supplementary Fig. S3A), and G_2_/M-phase arrest (Supplementary Fig. S3B-D). Moreover, GSK126 diminished the levels of H3K27me3 and increased the protein levels of p53 and p16 (Supplementary Fig. S3E). The activation of p53 was further assessed by transcription of p53 target genes such as *p15* and *p16* (Supplementary Fig. S3F). These results suggest that GSK126 leads to declined H3K27me3 and activation of p53.

### EZH2 inactivation by GSK126 induces apoptosis and abrogates outgrowth of UM tumor

**Fig. 3:**
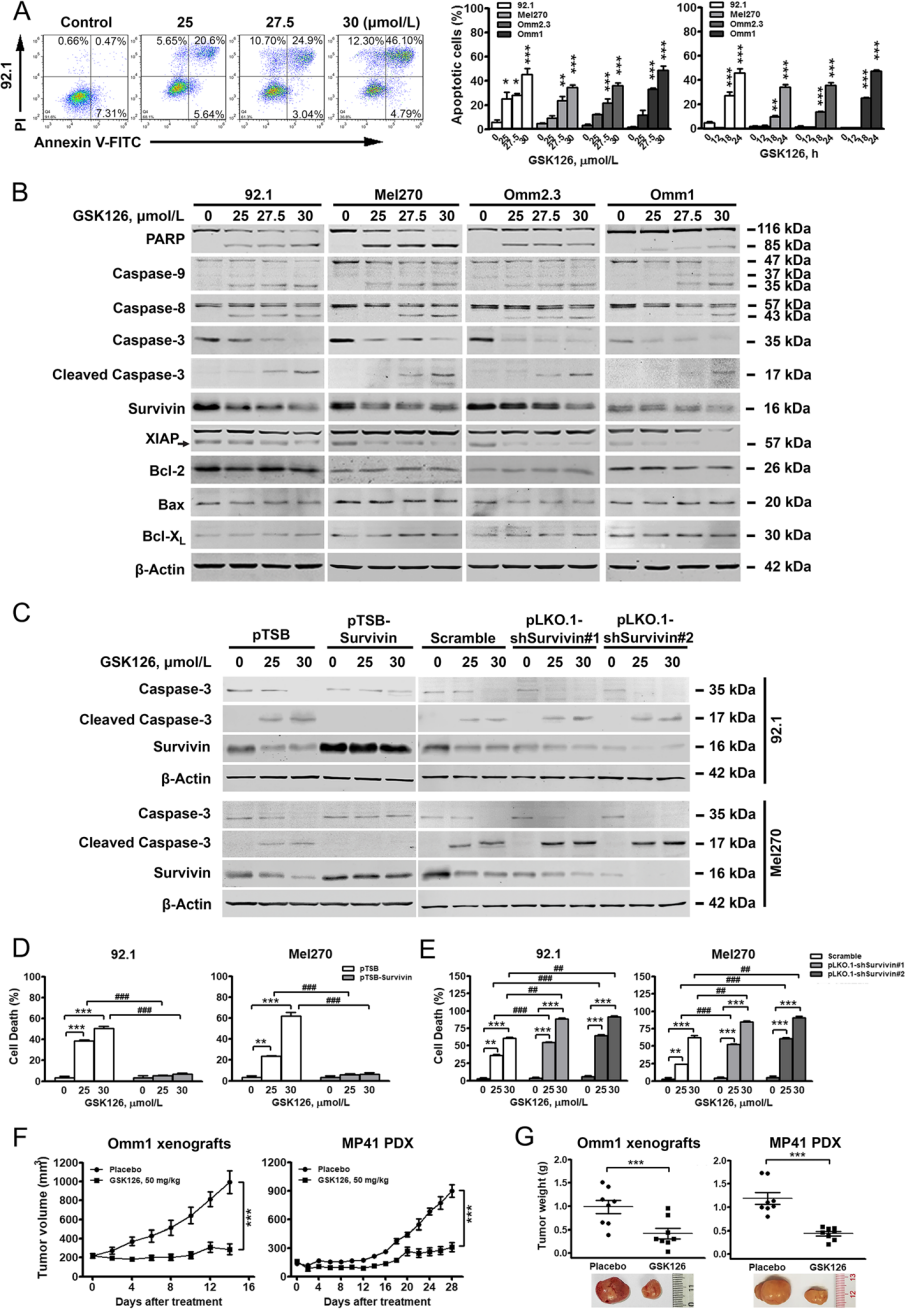
EZH2 inactivation by GSK126 induces apoptosis and abrogates outgrowth of UM tumor. **a-b** GSK126 induced apoptosis in UM cells. UM cells were treated with the indicating concentrations of GSK126 for 24 h, followed by staining with Annexin V-FITC and PI for flow cytometer analysis. A set of representative dot plots (*left*) and bar charts of annexin-V^+^ cell percentage (*middle and right*) from 3 independent experiments were shown. The *y*-axis represents the sum of the top right and bottom right quadrants. *, *P* < 0.05; **, *P* < 0.01; ***, *P* < 0.0001, one-way ANOVA, *post hoc* intergroup comparisons (**a**). Western blotting analysis of PARP cleavage, activation of caspase-9, − 8, − 3 and apoptosis-related proteins in GSK126 treated UM cells were shown (**b**). **c-e** Survivin mediated GSK126-induced cell apoptosis in UM. Stably expressed with plasmids or shRNAs as indicated, 92.1 and Mel270 cells were incubated with GSK126 for 24 h, followed by cell death detection. Western blotting analysis of cleaved caspase-3 (**c**) and Trypan blue exclusion assay were applied (**d** and **e**). **, *P* < 0.01; ***, *P* < 0.0001, one-way ANOVA, *post hoc* intergroup comparisons. ##, *P* < 0.01; ###, *P* < 0.0001, Student’s *t* test. **f-g** GSK126 abrogated outgrowth of xenografted Omm1 tumor and MP41 PDX in NOD/SCID mice. The estimated tumor volumes were plotted against the day of treatment. The last measurements of the 2 groups were compared (**f**). Weight of tumors dissected from the mice treated with placebo and GSK126 was compared (**g**). ***, *P* < 0.0001, Student’s *t* test

We next evaluated GSK126-induced apoptosis in UM. GSK126 significantly increased cell death in UM cells (Fig. 3a). Specific cleavage of PARP and activation of caspase-9, − 8 and − 3 were in a concentration- (Fig. 3b) and time-dependent (Supplementary Fig. S4A) manner. Apoptosis-related proteins in whole cell lysates showed a decrease in levels of Survivin and XIAP in the GSK126-treated UM cells (Fig. 3b and Supplementary Fig. S4A). Notably, the release of cytochrome *c* and AIF in the cytosolic fraction was detected (Supplementary Fig. S4B). These findings suggest that GSK126 induces apoptosis in UM cells by triggering the intrinsic pathway.

Because Survivin is a key factor mediating UM apoptosis [17], we evaluated the significance of Survivin in apoptosis induced by GSK126. Ectopic overexpression of Survivin significantly attenuated GSK126-induced apoptosis (Fig. 3c and d). Survivin silence alone was insufficient to induce apoptosis. However, downregulation of Survivin apparently increased the sensitivity of UM cells to GSK126-induced apoptosis (Fig. 3c and e). Our findings indicate that Survivin plays a vital role in GSK126-induced apoptosis in UM cells.

### GSK126 inhibits outgrowth of xenografted UM tumors and UM patient-derived xenografts (PDX) in NOD/SCID mice

To investigate the *in vivo* inhibitory effects of GSK126*,* the subcutaneous xenografted mouse model was employed [6]. The tumor volumes and weights from the GSK126-treated mice were significantly diminished (Fig. 3f-g, *left*). IHC analysis of Ki67 further showed reduced proliferation with GSK126 (Supplementary Fig. S4C, *left*). Meanwhile, GSK126 induced apoptosis *in vivo* as indicated by TUNEL assay (Supplementary Fig. S4D, *left*).

To better mimic the response of UM patients to GSK126, NOD/SCID mice with UM PDX were administrated with GSK126. Similar results of those obtained in Omm1 xenografted mice treated with GSK126 were observed (Fig. 3f-g, Supplementary Fig. S4C-D, *right*). In aggregate, the results indicate that GSK126 inhibits the *in vivo* outgrowth of UM.

### ALDH identifies a subpopulation enriched for CSCs in UM

**Fig. 4:**
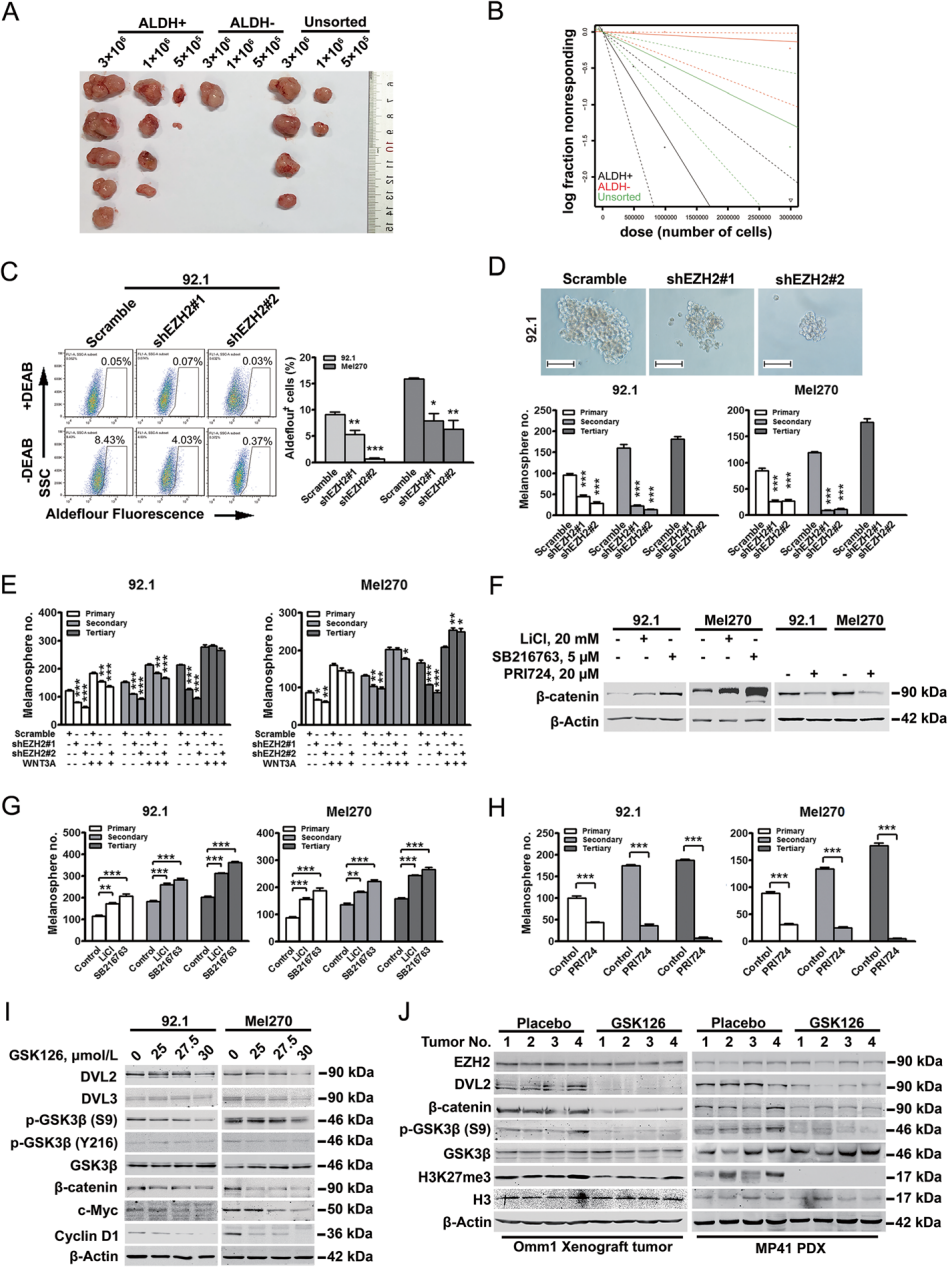
EZH2 confers maintenance of CSCs in UM involving Wnt/β-Catenin signaling. **a-b** ALDH^+^ subpopulation was enriched with CSCs in UM. Sorted ALDH^+^, ALDH^−^ Omm1 subpopulation and unsorted Omm1 cells were incubated subcutaneously for limiting dilution assay (**a**). CSC frequency was analyzed by Poisson statistics (**b**). **c** Depletion of EZH2 reduced the ALDH^+^ subpopulation. A set of representative scatter plot of ALDH assay by the flow cytometry analysis were shown. DEAB treatment is applied as a negative control for ALDH^+^ gating. Representative plot and bar charts from 3 independent experiments were shown. *, *P* < 0.05; **, *P* < 0.01; ***, *P* < 0.0001, one-way ANOVA, *post hoc* intergroup comparisons. **d** Transduction by lentiviral shRNA against EZH2 prohibited melanosphere formation and serially-replating ability. Representative photos of melanospheres (*top*) and bar charts of melanosphere numbers (*bottom*) were shown. ***, *P* < 0.0001, one-way ANOVA, *post hoc* intergroup comparisons. **e** WNT3A recombinant boosted the melanosphere formation and serially-replating ability. 92.1 and Mel270 cells were cultured with or without WNT3A (20 ng/mL) for melanosphere formation. *, *P* < 0.05; **, *P* < 0.01; ***, *P* < 0.0001, one-way ANOVA, *post hoc* intergroup comparisons. **f-h** Manipulation of Wnt/signaling by inhibitors regulated melanosphere formation in UM. After incubated with GSK3β inhibitors LiCl or SB216763 as indicated for 24 h, 92.1 and Mel270 cells were subjected to Western blotting analysis (**f**) and melanosphere formation culture (**g**). Treatment with β-catenin inhibitor PRI724, Western blotting analysis (**f**) and melanosphere formation culture were applied (**h**). **, *P* < 0.01; ***, *P* < 0.0001, one-way ANOVA, *post hoc* intergroup comparisons. **i-j** GSK126 interdicted Wnt/β-catenin signaling *in vitro* and *in vivo*. 92.1 and Mel270 cells were treated with GSK126 as indicated for 24 h for Western blotting analysis (**i**). Omm1 subcutaneously xenografted and MP41 PDX tumors of NOD/SCID mice treated with placebo or GSK126 were subjected to Western blotting analysis with antibodies as indicated (**j**)

Despite of unavailability of specific biomarkers for CSCs in UM, the existence of CSCs in UM was extensively reported [6, 12, 18, 19]. ALDH is a widely acknowledged biomarker for CSCs in solid tumors [7, 20]. We hypothesized ALDH as a potential marker for CSC enrichment in UM. The proportion of ALDH^+^ population in Omm1 cells which can form subcutaneous xenografted tumors in NOD/SCID mice [6, 12] was ~ 5.6% (Supplementary Fig. S5A). Because limiting dilution assay is the best tumorigenicity method that commonly used for evaluation of CSCs [21], sorted ALDH^+^, ALDH^−^ and unsorted Omm1 cells were then subjected to limiting dilution assay (Supplementary Fig. S5A). Obviously, the ALDH^+^ Omm1 subpopulation formed more xenografted tumors than either ALDH^−^ Omm1 subpopulation or unsorted Omm1 cells (Fig. 4a). The frequency of CSCs was 1/710,687 in ALDH^+^ Omm1 subpopulation, 1/22,591,981 in ALDH^−^ Omm1 subpopulation; and 1/2,366,723 in unsorted Omm1 cell population, respectively (Fig. 4b and Supplementary Table S3). These data indicate that ALDH^+^ Omm1 subpopulation enriches CSCs in UM.

### EZH2 confers maintenance of CSCs in UM involving Wnt/β-catenin signaling

To elucidate whether EZH2 conferred maintenance of CSCs in UM, ALDH assay was performed. Depletion of EZH2 either by shRNAs or GSK126 significantly reduced the ALDH^+^ percentage (Fig. 4c and Supplementary Fig. S5B). Moreover, the *in vivo* limiting dilution assay showed that inhibition of EZH2 eradicates CSCs in UM (Supplementary Table S4). As a functional indicator, the self-renewal capacity reflected by melanosphere formation and their serially-replating ability in EZH2-depleted-92.1 and -Mel270 cells were significantly compromised (Fig. 4d). Similar results were obtained in the GSK126-treated UM cells (Supplementary Fig. S5C).

Considering that EZH2-Wnt/β-catenin axis is found in CSCs of hepatocellular carcinoma [22], we sought the role of Wnt/β-catenin signaling in UM CSCs. Recombinant WNT3A boosted the abilities of melanosphere formation and serially-replating in scramble-UM cells and reconciled the self-renewal capability in EZH2-depleted UM cells (Fig. 4e). The positive effect of WNT3A on CSCs self-renewal may due to the enhancement of transcription of Wnt target genes (Supplementary Fig. S5D). In another approach, treatment with GSK3β inhibitors (e.g., LiCl and SB216763) gave rise to the accumulation of β-catenin and promoted serially-replating ability of melanosphere (Fig. 4f-g). In contrast, inhibition of Wnt/β-catenin signaling by PRI724 [23], hampered serial-replating ability of melanosphere (Fig. 4f, h and Supplementary Fig. S5E).

GSK126 treatment downregulated the protein levels of DVL2, β-catenin and its target genes such as c-Myc and Cyclin D1 (Fig. 4i and Supplementary Fig. S5F). Notably, GSK126 blocked the *in vivo* Wnt/β-catenin signaling as shown by Western blotting analysis of Omm1 xenografted and MP41 PDX tumors (Fig. 4j).

Collectively, these data indicate that EZH2 strengths the stemness of CSCs in UM through Wnt/β-catenin pathway.

### EZH2 increases DVL2 expression through suppressing miR-29c

DVL is a critical mediator of Wnt signaling transduction [24]. Because DVL1 is not detectable in UM (data not shown), and because GSK126 downregulated DVL2 expression, we further sought to unveil the underlying mechanism by which EZH2 regulated DVL2. The effect of EZH2 in propelling Wnt/β-catenin signaling was firstly confirmed (Supplementary Fig. S6A). The protein levels of DVL2 were barely altered while mRNA levels of *DVL2* were significantly declined (Supplementary Fig. S6B-D), indicating that the DVL2 regulation by EZH2 may occur predominantly at transcription level.

Because DVL2 transcription is suppressed by miR-29c-3p which can be epigenetically repressed by EZH2 recruitment on its promoter [25], we examined whether EZH2 increased DVL2 expression by suppressing miR-29c-3p in UM cells. Inhibitor of miR-29c-3p elevated mRNA and protein levels of *DVL2* (Supplementary Fig. S6E). Conversely, mimic of miR-29c-3p downregulated the mRNA and protein levels of *DVL2* in Mel270 cells (Supplementary Fig. S6E), verifying the transcription involvement of miR-29c-3p in *DVL2*.

Inhibition of EZH2 markedly augmented the expression of mature *miR-29c-3p* (Supplementary Fig. S6F-G). ChIP assay further revealed the recruitment of EZH2 (Supplementary Fig. S6H) and H3K27me3 (Supplementary Fig. S6I) to *miR-29b2/c* gene promoter, which was obviously diminished in the EZH2-depleted UM cells (Supplementary Fig. S6I-J). Forced re-expression of EZH2 in the EZH2-silenced rescued such recruitment (Supplementary Fig. S6H-I). Because EZH2 may serve as a recruitment platform for DNA methyltransferases (DNMTs) especially DNMT3A for gene silencing [26], we next ascertained whether DNMT3A was also recruited to *miR-29b2/c* gene promoter (Supplementary Fig. S6K). This enrichment was markedly abolished with EZH2 silencing, which was, however, rescued by forced re-expression of EZH2 (Supplementary Fig. S6K). Consistently, SGI-1027, a DNMT3A inhibitor, significantly upregulated of *miR-29c-3p* mRNA (Supplementary Fig. S6L). Taken together, these data demonstrate that EZH2 enhances DVL2 transcription by DNMT3-dependent re-expression of miR-29c.

### EZH2 augments migration and invasion of UM cells

**Fig. 5:**
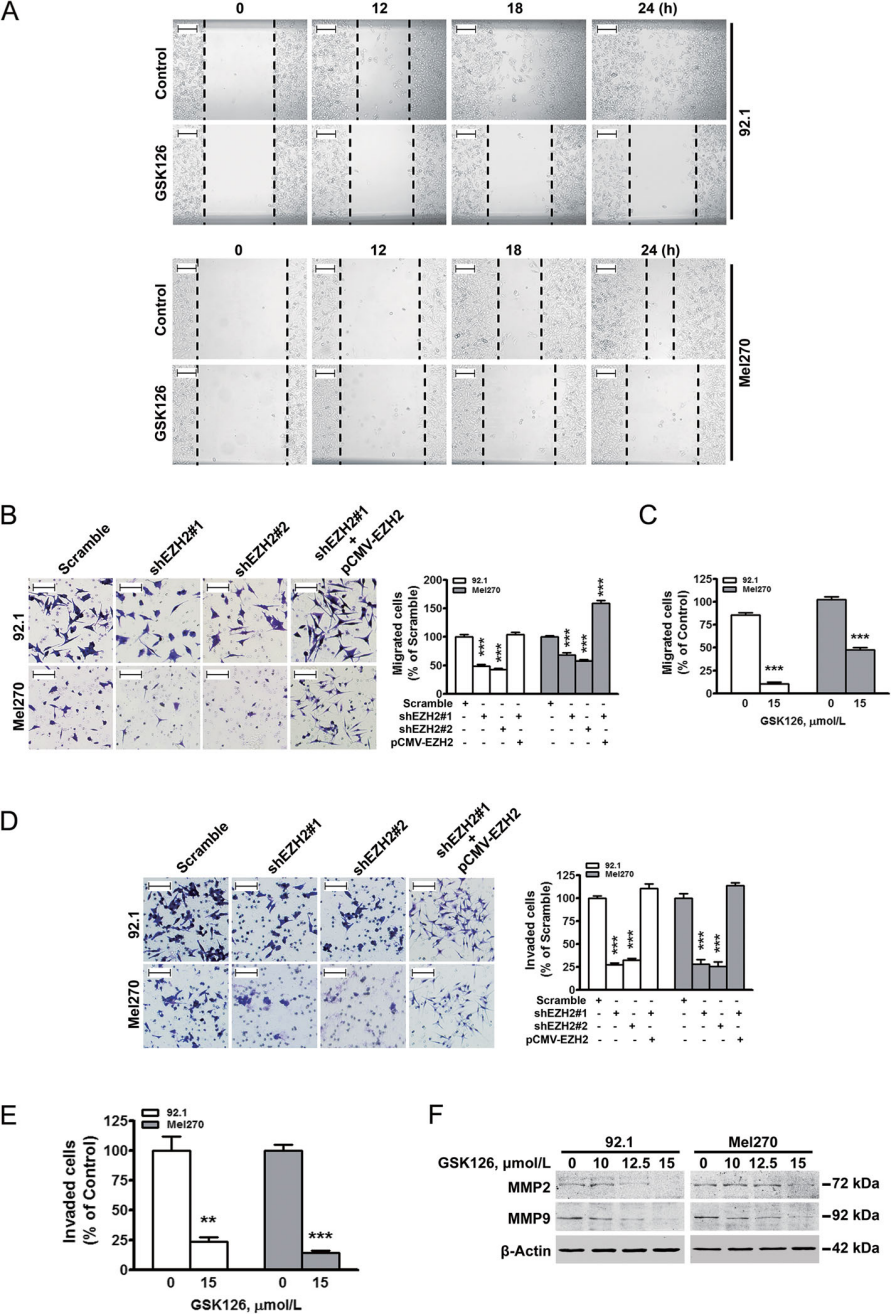
EZH2 loss suppresses migration and invasion of UM cells. **a** GSK126 inhibited the wound healing ability in UM cells. 92.1 and Mel270 cells were pre-treated with 15.0 μmol/L of GSK126, and then subjected to wound healing assay. Photos were taken in the same field at the indicating time. Original magnification, 100× (scale bar, 100 μm), Olympus IX71. **b-c** Depletion of EZH2 suppressed the migration of UM cells. 92.1 and Mel270 cells transfected with Scramble, or lentiviral shEZH2 in the absence or presence of EZH2-encoding constructs were subjected to transwell assay (**b**, *left*); the number of migrated cells was normalized relative to scramble (**b**, *right*). Data of bar charts were from 3 random fields. ***, *P* < 0.0001, one-way ANOVA, *post hoc* intergroup comparisons. After pre-treated with 15.0 μmol/L GSK126 for 24 h, 92.1 and Mel270 cells were evaluated for migration ability by transwell assay (**c**). Data of bar charts were from 3 random fields. ***, *P* < 0.0001, Student’s *t* test. **d-f** Depletion of EZH2 suppressed the invasion of UM cells. 92.1 and Mel270 cells transfected with Scramble, or lentiviral shEZH2 in the absence or presence of EZH2-encoding constructs were subjected to transwell invasion assay (**d**, *left*); the number of the invaded cells was normalized relative to scramble (**d**, *right*). Data of bar charts were from 3 random fields. ***, *P* < 0.0001, one-way ANOVA, *post hoc* intergroup comparisons. After treated with 15.0 μmol/L GSK126 for 24 h, 92.1 and Mel270 cells were seeded for transwell invasion assay (**e**). Data of bar charts were from 3 random fields. **, *P* < 0.01; ***, *P* < 0.0001, Student’s *t* test. 92.1 and Mel270 cells were treated with GSK126 as indicated for 24 h, followed by Western blotting analysis for MMP2 and MMP9 (**f**)

To validate the function of EZH2 in mediating the dissemination of UM cells, we examined the migration and invasion. GSK126 suppressed the wound-healing ability (Fig. 5a). Depletion of EZH2 resulted in a dramatically decreased migration (Fig. 5b-c). When EZH2 was reconstituted, the decrease tendency of migration was, nonetheless, reversed (Fig. 5b). Matrigel-coated transwell assay revealed that the invasion ability of EZH2-depleted UM cells was significantly weakened, which was restored with EZH2 refill (Fig. 5d-e). The expression of matrix metalloproteinase 9 (MMP9) and MMP2, critical metastasis-associated proteins, was reduced in the GSK126-treated UM cells (Fig. 5f). Collectively, these data suggest that EZH2 strengthens migration and invasion in UM, which can be blocked by EZH2 inhibition.

### EZH2 facilitates motility of UM cells via RhoGDIγ-Rac1 axis

Cell motility is one of the determinant causes of migration and invasion. We next investigated the polymerization of F-actin. Dimmer fluorescence signal of F-actin was observed in EZH2-depleted UM cells. With EZH2 refill, the increased fluorescence intensity of F-actin, the appearance of membrane ruffles and the formation of lamellipodia were regained (Supplementary Fig. S7A-B). These results indicate that EZH2 facilitates the UM cell motility through inducing F-actin polymerization.

We next evaluated Rho GTPases, important regulators of actin cytoskeletal organization and cell motility [27]. Pull-down assay showed a substantially decreased level of active-Rac1 protein, a member of the Rho GTPase in EZH2-silenced Mel270 cells, relative to the scramble control (Supplementary Fig. S7C). Re-expression of EZH2 in the EZH2-silenced Mel270 cells restored active-Rac1 (Supplementary Fig. S7C). Consistently, the downstream signaling of Rac1 was blocked with EZH2 depletion; but restored when EZH2 was reconstituted in EZH2-silenced UM cells (Supplementary Fig. S7D). These results suggest that EZH2 activates Rho GTPase.

Further analysis showed that knockdown of EZH2 increased the levels of Rho GTPase-inhibitory RhoGDIγ (Supplementary Fig. S7D). When EZH2 was reconstituted in EZH2-silenced UM cells, the expression of RhoGDIγ was, nevertheless, declined to the level comparable to that in the Scramble cells (Supplementary Fig. S7D). qRT-PCR analysis showed that the mRNA levels of *ARHGDIG* were significantly ascended in the EZH2-silenced UM cells (Supplementary Fig. S7E, *left*) and GSK126-treated UM cells (Supplementary Fig. S7E, *right*).

We next asked why EZH2 repressed transcription of *ARHGDIG* gene. ChIP assay revealed EZH2 recruitment on the promoter of *ARHGDIG* gene in the Scramble-treated UM cells (Supplementary Fig. S7F). Silence of EZH2 abrogated such EZH2 recruitment, however, the reconstitution of EZH2 in EZH2-silenced UM cells reconducted EZH2 recruitment (Supplementary Fig. S7F). To more directly interrogate the transcription repression on *ARHGDIG*, H3K27me3 antibody was also utilized to perform ChIP assay. The binding of H3K27me3 to the promoter of *ARHGDIG* was abolished with EZH2 depletion, which was rescued when EZH2 was reconstituted (Supplementary Fig. S7G). The pharmacological inhibition of EZH2 by GSK126 revealed the suppression of binding of H3K27me3 to *ARHGDIG* promoter (Supplementary Fig. S7H).

Since DNMT3 is a widely existed executor to directly silence transcription of target genes in epigenetic complex, we further examined whether DNMT3A was involved. ChIP assay showed enrichment of DNMT3A at the promoter of *ARHGDIG* in Scramble-cells*.* Depletion of EZH2 decreased such enrichment, while re-expression of EZH2 rescued this enrichment (Supplementary Fig. S7I). If DNMT3A worked in the transcriptional complex, it was postulated that inhibition of DNMT3A would release the transcription of *ARHGDIG* gene. Indeed, the mRNA levels of *ARHGDIG* were significantly increased when the UM cells were treated with SGI-1027 (Supplementary Fig. S7J). Together, these data suggest that EZH2 may facilitate migration and invasion of UM cells via RhoGDIγ-Rac1 axis.

### Depletion of EZH2 attenuates the liver metastasis of UM

**Fig. 6:**
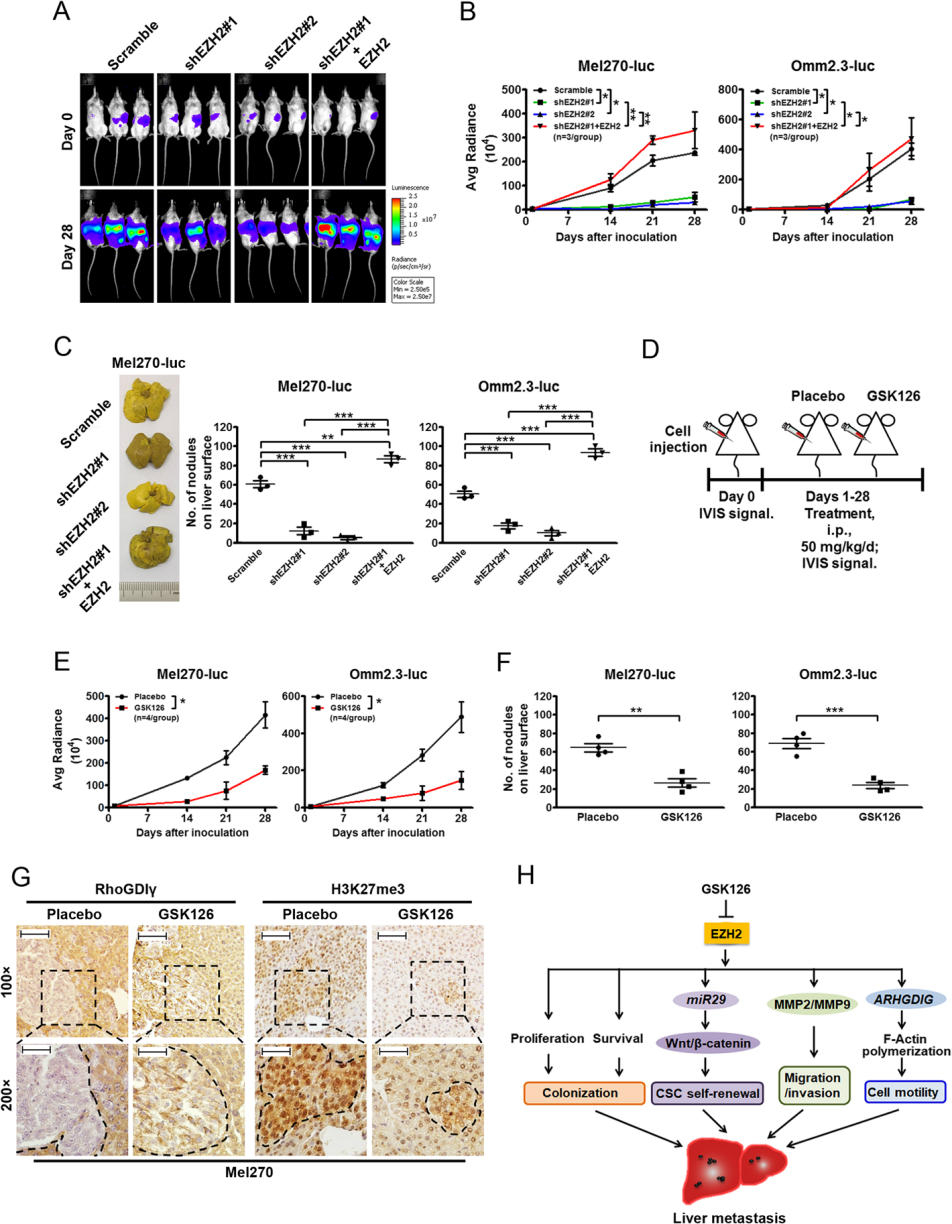
Depletion of EZH2 abrogates the liver metastasis of UM in mice. **a-c** Silencing of EZH2 attenuated liver metastasis of UM in NOG mice. Mel270-luc and Omm2.3-luc cells transduced with Scramble or lentiviral shEZH2 were intrasplenically injected into NOG mice. IVIS signals of luciferase activity of Mel270-luc on day 0 and day 28 were shown (**a**). The curves were IVIS signals against days after inoculation (**b**). *, *P* < 0.05; **, *P* < 0.01, one-way ANOVA, *post hoc* intergroup comparisons. Photos of livers fixed in Bouin’s solution were shown (**c**, *left*) and numbers of nodules on liver surface were counted (**c**, *middle* and *right*). Each dot represents one mouse. **, *P* < 0.01; ***, *P* < 0.0001, one-way ANOVA, *post hoc* intergroup comparisons. **d-g** Pharmacologic inhibition of EZH2 attenuated the metastasis of UM cells. NOG mice were intrasplenically injected with Mel270-luc or Omm2.3-Luc cells and administrated with GSK126 (50 mg/kg/d, i.p.) for 4 weeks (**d**). The IVIS signals were recorded (**e**). *, *P* < 0.05; Student’s *t* test. The metastatic tumor nodules on the liver surface were counted (**f**). **, *P* < 0.01; ***, *P* < 0.0001, Student’s *t* test. IHC staining of RhoGDIγ and H3K27me3 in metastatic liver sections from Mel270-luc-bearing NOG mice after treatment of placebo or GSK126 (**g**). Photos were recorded by Olympus IX71. Original magnification was 100× (Scale bar, 100 μm) and 200× (Scale bar, 50 μm). **h** A proposed model of EZH2 in orchestrating the metastasis of UM

We next examined whether targeting EZH2 efficiently abrogated liver metastasis in UM. We employed a hepatic metastasis model using NOG mice intrasplenically injected with Scramble- or EZH2-silenced UM cells [6]. The status of EZH2 expression was initially verified before cell inoculation (Supplementary Fig. S8A). EZH2 silence appreciably attenuated the metastatic tumor burdens in liver, while re-expression with EZH2 conspicuously increased the burdens (Fig. 6a-c). H&E staining analysis of liver sections showed similar results (Supplementary Fig. S8B). In an alternative approach, we transplanted UM cells into spleens of NOG mice, and then administrated the mice with either placebo or GSK126 (Fig. 6d). GSK126 treatment substantially reduced the IVIS signals (Fig. 6e) and the liver metastatic nodule numbers (Fig. 6f and Supplementary Fig. S8C). Additionally, IHC staining revealed that the expression of RhoGDIγ in the metastatic liver sections with GSK126 was expanded comparing with those with placebo, while levels of H3K27me3 were declined in those sections from GSK126-treated mice (Fig. 6g and Supplementary Fig. S8D). These data further validate that EZH2 facilitates the UM metastasis in liver and might be a potent target for metastatic UM patients.

### EZH2 and BAP1 are two independent factors in UM

The regulation of BAP1 in epigenetic switch in UM prompt us to unveil the underlying correlation of BAP1 and EZH2 [28]. The expression of EZH2 and its HMT indicator H3K27me3 was not altered in UM cells transfected with BAP1-WT or its kinase dead mutation BAP1-C91S, comparing with that in vector-transfected-UM cells (Supplementary Fig. S9A). Reciprocally, the BAP1 expression in stably shEZH2-silenced UM cells was not altered (Supplementary Fig. S9B). These results revealed that EZH2 may be a target independent of BAP1 in UM.

## Discussion

Hepatic metastasis was observed in patients with UM even 15 years after successful management of primary tumor [2]. The long latency may partially reflect the additional time needed to perform the adaptive liver-specific program and offer opportunities of intervention. We here demonstrated that EZH2 overexpressed in UM promoted the *in vitro* and *in vivo* growth of UM; increased the percentage and self-renewal of CSCs and augmented migration and invasion of UM cells. Targeting EZH2 either by genetics or small molecule inhibitor decreased CSCs and motility and abrogated the liver metastasis of UM.

Gain of stemness of CTCs is presumed to be an essential prerequisite to their successful colonization at the distant tissues [1]. Because no putative biomarkers are available for UM CSCs [19], we first validated that ALDH^+^ Omm1 cells are a CSCs-enriched subpopulation in UM. Further, we evaluated the functional features of CSCs in UM by analyzing the melanosphere formation and serially-replating capability and the in vivo limiting dilution assay for the frequency of CSCs. Our results are consistent with the findings that EZH2 expression is elevated in CSC subpopulation and EZH2 promotes the expansion of CSCs in breast and colorectal cancer [29, 30].

Among the development-related pathways (e.g., Wnt/β-catenin, HHg, Notch), Wnt/β-catenin pathway is essential for maintenance of CSCs by regulating their self-renewal in multiple types of cancer (e.g., melanoma, glioblastoma, colon cancer and breast cancer) [8, 30, 31]. Likewise, Wnt/β-catenin pathway is critical for the CSCs of UM as illustrated in the present study. It is universal that EZH2 positively regulates Wnt/β-catenin signaling in hepatocellular carcinoma. The underlying mechanisms involve: 1) EZH2 activates Wnt/β-catenin signaling by promoting the methylation of β-catenin resulting in inhibiting its ubiquitination and facilitating the stability of β-catenin [22]; 2) High EZH2 occupancy and reduced expression of Wnt antagonists were found to constitutively activate of Wnt/β-catenin signaling [32]; 3) The present data identifying an upstream regulator miR-29c on *DVL2* expression in UM extend these findings, and further improves the understanding how EZH2 affect Wnt/β-catenin pathway.

The downstream target genes and pathways of repressive H3K27me3 histone modification by EZH2 are multiple. Wnt/β-catenin signaling pathway is one of the important mediators for CSC regulation by EZH2. Other downstream target genes and pathways cannot be excluded.

The whole metastasis process requires an essential capability namely motility acquisition powered by polymerization of actin filaments (F-actin) [33]. We found that EZH2 accelerates the migration and invasion which are motivated by F-actin polymerization, in the metastatic cascade of UM cells. These findings are in line with the previous report that EZH2 regulates actin polymerization in prostate cancer [34]. Loss of RhoGDIγ expression is associated with poor prognosis and promotes metastasis and invasion in hepatocellular carcinoma [35]. Our results indicate that EZH2 is a key arbiter mediating cell motility through RhoGDIγ. These results are consistent with previous reports that RhoGDIγ preventing neural stem cell migration through Rac1 [36].

RhoGDIγ is a poorly characterized member of the RhoGDI family. Several transcription factors are able to bind to the promoter of *ARHGDIG* [37], with the underlying mechanism unveiled. EZH2 silencing or pharmacological inhibition of EZH2 further resulted in reduction of enrichment of H3K27me3 and DNMT3A at the promoter of *ARHGDIG*. Our findings may shed light on the mechanism of *ARHGDIG* expression.

We also asked why EZH2 is overexpressed in UM. Because Cyclin D1 is overexpressed in patients with UM [38], we examined whether E2F1 involved in the EZH2 overexpression in UM. The outcome confirmed that E2F1 is predominantly elevated in UM tissues and cells, and that overexpressed E2F1 promotes transcription of EZH2 gene. Findings from us and other groups indicate that E2F1 may directly bind to the promoter of EZH2 gene and increase transcription [39]. Other mechanisms including gain of copy number which is found in ~ 10% of UM patients in TCGA database cannot be excluded.

### ALDH is a valid biomarker for CSCs in UM

CSCs are those tumor cells with initiating ability, self-renewal potential, and intrinsic resistance to conventional therapeutics. Serial in vitro tumorsphere formation is usually a surrogate assay for self-renewal; the gold standard for defining CSCs has been serial in vivo transplantation [40]. Doherty RE et al. claimed that the classical hierarchy model of CSCs was challenged based on their proliferation capacity of the sorted subpopulations of UM cells with conventional colony formation assay [41]. However, the conventional clonogenic assay is actually insufficient to reflect the features of CSCs. No functional parameters (e.g., serial tumorsphere/replating, in vivo tumorigenicity by limiting dilution) were included in their study [41], which are minimum criteria for CSC evaluation.

In addition to the biomarker, we evaluated CSCs in UM by functional parameters (melanosphere/replating capacity and in vivo CSCs frequency assessed by limiting dilution assay). Our results, however, showed that the sorted ALDH^+^ Omm1 cells displayed ~ 30- and ~ 3-fold increase in the in vivo tumorigenicity of CSCs when compared with ALDH^−^ subpopulation and unsorted Omm1 cells, respectively. Our results support that ALDH is a valid marker for CSCs in UM, as vastly reported in the other cancer types such as cutaneous melanoma [42], breast cancer [7], prostate cancer [43] and lung adenocarcinoma [44].

### EZH2, BAP1 and other prognostic factors in UM

Based on cytogenetic and genetic analysis in UM patient samples, several predictors for metastatic risk were identified: monosomy 3, BAP1 and Class1 vs 2 GEP prognostic factors [5, 45–47]. The optimal three-gene sets (PHLDA1, FZD6, and ENPP2) which can predict class label (Class 1 vs 2 GEP) in UM patients were applied for comparison [47]. These factors are of great value in predicting the metastatic risk and guiding the treatment in UM patients (Supplementary Fig. S1). However, these factors are difficult to be targeted for therapy. Because the original GEP (gene expression profiling) dataset for classification was not uploaded by the authors in the original paper, we cannot further analyze whether EZH2 expression is enhanced in class 2 vs class 1 uveal melanomas [46], and whether EZH2 is a therapeutic target for inhibiting the metastasis to the liver of class II tumors. Further study with more samples is needed in future.

Loss-of-function mutations of the *BAP1* gene are found in ~ 80% of metastatic UM patients, rendering BAP1 an independent indicator for UM metastasis [5]. Nevertheless, the key node of BAP1 pathway in driving or mediating UM metastasis remains unexplored.

LaFave LM et al. reported that knockout of BAP1 results in elevated transcription of *EZH2* gene in acute myeloid leukemia mouse bone marrow cells and human mesothelioma cells [48]. However, our results indicate that BAP1 pathway and EZH2 seem to be two independent therapeutic targets in UM. The discrepancy of BAP1 in regulating EZH2 in human and murine cells may be cell type context-dependent.

The significance of EZH2 in predicting overall survival in TCGA database suggested that EZH2 is an important prognostic factor. However, further validation of EZH2 in patient samples with clinical follow-up data remains to be done. The more valuable role of EZH2 in UM according to the present genetic and pharmacological studies is that EZH2 may be a potent therapeutic target in UM (Fig. 6h).

### EZH2 is a druggable target in UM

Pharmacological inhibition of EZH2, the core enzymatic component of PRC2, has been illustrated to effectively kill various cancer cells [49]. The inhibitory potency of EZH2 inhibitors on UM cell viability is correlated with the magnitude of the inhibitory effect of EZH2 inhibitors on lysine methylation activity, revealing that EZH2 is an effective target. Comparing to the other 5 HMT inhibitors, EPZ6438 manifested the least inhibitory potency against both EZH2 enzyme activity and cell viability with relatively higher IC_50_ values (77.83–92.75 μmol/L). Although Schoumacher M et al. claimed in the title that uveal melanoma cells are resistant to EZH2 inhibition based on a single EZH2 inhibitor, two cell lines i.e. 92.1 and MP46 exhibited a decrease of approximately 35% and 65% in colony growth [50]. Their data contradicted their title by themselves.

In contrast to the simple readout evaluation (i.e., proliferation and *in vitro* only) [50] in the correspondence to editor by Schoumacher M et al., the *in vivo* metastasis and stemness for readout which are more appropriate endpoints of epigenetic reprogramming were included in our study. Our *in vitro* and *in vivo* data consistently revealed that the capacities of clonogenicity, stemness, and metastasis were significantly reduced in UM after treatment with EZH2 shRNAs or GSK126, suggesting that EZH2 is an effective druggable target.

The intra-splenic injection model generally represents the late stages of the extravasation and colonization in the host organ and failed to reflect the early stages of local invasion and intravasation in the metastatic process. How EZH2 exerts its role in the early stages of metastatic process needs further investigation. Collectively, our findings support that EZH2 facilitates metastasis of UM, and that EZH2 may be a promising druggable target for UM patients.

## Conclusions

In summary, we found that EZH2 was overexpressed in UM and promoted the growth of UM. EZH2 increased the percentage and self-renewal of CSCs and facilitated migration and invasion of UM cells. Targeting EZH2 abrogated the liver metastasis of UM. These findings validate EZH2 as a druggable target in metastatic UM patients and shed light on the understanding and interfering the complicated metastatic process.

## Supplementary information

**Additional file 1 Supplementary Table S1**. The association between EZH2 expression and clinicopathologic features in UM. **Supplementary Table S2**. IC50 values of HMT inhibitors, μmol/L. **Supplementary Table S3**. Limiting dilution analysis in NOD/SCID mice with ALDH+, ALDH- or unsorted Omm1 cells. **Supplementary Table S4**. Limiting dilution analysis in NOD/SCID mice with or without GSK126 treatment. **Supplementary Table S5**. Primers for ChIP and qPCR.

**Additional file 2 Supplementary Figure S1**. The prognostic factors of EZH2, BAP1, and Class 1 vs 2 GEP in the correlation of UM overall survival and metastasis-free survival in published database. **Supplementary Figure S2**. Verification of EZH2 overexpression and knockdown in UM cells. **Supplementary Figure S3**. Pharmacologic inhibition of EZH2 induces G_2_/M phase arrest and activation of p53. **Supplementary Figure S4**. GSK126 induces apoptosis in UM via triggering intrinsic pathway. **Supplementary Figure S5**. EZH2 confers maintenance of cancer stem cells (CSCs) in uveal melanoma involing Wnt/β-catenin signaling. **Supplementary Figure S6**. EZH2 confers maintenance of cancer stem cells via suppressing miR-29b2/c gene transcription in uveal melanoma. **Supplementary Figure S7**. EZH2 mediates motility of UM cells via RhoGDIγ-Rac1 axis. **Supplemetary Figure S8**. EZH2 facilities liver metastasis of UM in NOG mice. **Supplementary Figure S9**. The expression of BAP1 and EZH2 is parallel in UM cells.

**Additional file 3.** Supplementary Materials and Methods.

## Data Availability

All data generated or analyzed during the current study are included in this published article (and its supplementary files).

## Abbreviations

AIF: Apoptosis-inducing factor
ALDH: Aldehyde dehydrogenase
ChIP: Chromatin immunoprecipitation
CHX: Cycloheximide
COX II: Cytochrome *c* oxidase subunit II
CSC: Cancer stem-like cell
CTCs: Circulating tumor cells
DEAB: Diethylaminobenzaldehyde
DNMTs: DNA methyltransferases
EZH2: Enhancer of zeste homolog 2
F-actin: Actin filaments
FITC: Fluorescein isothiocyanate
H&E: Hematoxylin and eosin
HMT: Histone methyltransferase
IHC: Immunohistochemistry
IVIS: In vivo imaging system
MICs: Metastasis initiating cells
MMP: Matrix Metalloproteinase
PCNA: Proliferating cell nuclear antigen
PDX: Patient-derived xenograft
PI: Propidium iodide
PRC2: Polycomb repressive complex 2
UM: Uveal melanoma
