# Supplementary material for: Verification of EZH2 as a druggable target in metastatic uveal melanoma
Source: Mol Cancer. 2020 Mar 4;19:52. doi: 10.1186/s12943-020-01173-x (PMC7055080; doi:10.1186/s12943-020-01173-x)
Supplement: Supplementary file 1 — Additional file 1 Supplementary Table S1. The association between EZH2 expression and clinicopathologic features in UM. Supplementary Table S2. IC50 values of HMT inhibitors, μmol/L. Supplementary Table S3. Limiting dilution analysis in NOD/SCID mice with ALDH+, ALDH- or unsorted Omm1 cells. Supplementary Table S4. Limiting dilution analysis in NOD/SCID mice with or without GSK126 treatment. Supplementary Table S5. Primers for ChIP and qPCR. [file 12943_2020_1173_MOESM1_ESM.doc]

**Supplementary Table S1. The association between EZH2 expression and clinicopathologic features in UM.**

| **Variables** | **EZH2** | | | | |  |
| --- | --- | --- | --- | --- | --- | --- |
|  | **cases** | **Negative** | **Low** | **Medium** | **High** | **p Value§** |
| Age (y) |  |  |  |  |  | 0.8976 |
| ≤50*  >50 | 27  23 | 3  3 | 4  5 | 9  6 | 11  9 |  |
| Gender  Male  Female | 28  22 | 4  2 | 8  1 | 6  9 | 10  10 | 0.1078 |
| The largest basal diameter (mm)  ≤15  >15 | 36  14 | 5  1 | 6  3 | 15  0 | 10  10 | 0.0110 |
| Thickness (mm)  ≤11  >11 | 20  30 | 2  4 | 5  4 | 10  5 | 3  17 | 0.0136 |
| Ki67 expression level  ≤10%  >10% | 37  13 | 5  1 | 5  4 | 11  4 | 16  4 | 0.5240 |

* Average age; § Chi-square test was independently applied for each variable. The largest basal diameter (15 mm) and Thickness (11 mm) are medians of the cohort.

**Supplementary Table S2. IC_50_ values of HMT inhibitors, µmol/L.**

| **Cells** | **HMT inhibitors** | | | | | |
| --- | --- | --- | --- | --- | --- | --- |
|  | **GSK126** | **GSK503** | **UNC1999** | **EI1** | **CPI1205** | **EZP6438** |
| **92.1** | 7.83 | 17.77 | 13.67 | 55.52 | 51.52 | 92.75 |
| **Mel270** | 12.32 | 9.93 | 11.37 | 32.58 | 31.8 | 94.65 |
| **Omm2.3** | 11.99 | 9.68 | 7.52 | 47.75 | 44.54 | 78.19 |
| **Omm1** | 13.57 | 17.16 | 7.82 | 35.85 | 36.62 | 77.83 |

**Supplementary Table S3. Limiting dilution analysis in NOD/SCID mice with ALDH^+^, ALDH^-^ or unsorted Omm1 cells.**

| Cell dose | Engrafted/Tested | | |
| --- | --- | --- | --- |
|  | ALDH^+^ | ALDH^-^ | Unsorted |
| 3×10^6^ | 5/5 | 1/5 | 4/5 |
| 1×10^6^ | 4/5 | 0/5 | 2/5 |
| 5×10^5^ | 2/5 | 0/5 | 0/5 |
| CSC frequency | 1/710,687 | 1/22,591,981 | 1/2,366,723 |

**Supplementary Table S4. Limiting dilution analysis in NOD/SCID mice with or without GSK126 treatment**

| Cell dose | Engrafted/Tested | |
| --- | --- | --- |
|  | Control | GSK126 |
| 3×10^6^ | 8/8 | 0/8 |
| 1×10^6^ | 4/8 | 0/8 |
| 5×10^5^ | 3/8 | 0/8 |
| CSC frequency | 1/1,056,019 | 0 |

**Supplementary Table S5. Primers for ChIP and qPCR**

Primers for ChIP and qPCR

| Primers for ChIP (5'-3') | | |
| --- | --- | --- |
| *ARHGDIG* | Forward | CCAGGTCGCTAAGCCTTTGA |
|  | Reverse | CACCCTACAGGTTCACGGTC |
| *ARHGDIG -*Intron | Forward | CTCTAGCAGGTAGCCCCTGA |
|  | Reverse | TCCCTGACTCGGAGACCAAA |
| *ARHGDIG -*CDS | Forward | GGACCAGGTGTTTGTCCTGA |
|  | Reverse | TGGGAACAGAGGCATTAGGG |
| *DAB2IP* | Forward | CCTCCCAGTCACCTGTCATTT |
|  | Reverse | ATGGGAGACAGCTTCATTTGGG |
| Primers for qPCR (5'-3') | | |
| *EZH2* | Forward | GTACACGGGGATAGAGAATGTGG |
|  | Reverse | GGTGGGCGGCTTTCTTTATCA |
| *DVL2* | Forward | GAGGAAGAGACTCCCTACCTG |
|  | Reverse | CGGGCGTTGTCATCTGAAAT |
| *P53* | Forward | TCAACAAGATGTTTTGCCAACTG |
|  | Reverse | ATGTGCTGTGACTGCTTGTAGATG |
| *P21* | Forward | GACTCTCAGGGTCGAAAACGG |
|  | Reverse | GCGGATTAGGGCTTCCTCTT |
| *BAX* | Forward | GAACCATCATGGGCTGGACA |
|  | Reverse | GCGTCCCAAAGTAGGAGAGG |
| *NOXA* | Forward | GCAAGAACGCTCAACCGAG |
|  | Reverse | TTGAAGGAGTCCCCTCATGC |
| *PUMA* | Forward | ACCTCAACGCACAGTACGAG |
|  | Reverse | CGGGTGCAGGCACCTAATTG |
| *18S* | Forward | AAACGGCTACCACATCCAAG |
|  | Reverse | CCTCCAATGGATCCTCGTTA |
