# Supplementary material for: Verification of EZH2 as a druggable target in metastatic uveal melanoma
Source: Mol Cancer. 2020 Mar 4;19:52. doi: 10.1186/s12943-020-01173-x (PMC7055080; doi:10.1186/s12943-020-01173-x)
Supplement: Supplementary file 2 — Additional file 2 Supplementary Figure S1. The prognostic factors of EZH2, BAP1, and Class 1 vs 2 GEP in the correlation of UM overall survival and metastasis-free survival in published database. Supplementary Figure S2. Verification of EZH2 overexpression and knockdown in UM cells. Supplementary Figure S3. Pharmacologic inhibition of EZH2 induces G2/M phase arrest and activation of p53. Supplementary Figure S4. GSK126 induces apoptosis in UM via triggering intrinsic pathway. Supplementary Figure S5. EZH2 confers maintenance of cancer stem cells (CSCs) in uveal melanoma involing Wnt/β-catenin signaling. Supplementary Figure S6. EZH2 confers maintenance of cancer stem cells via suppressing miR-29b2/c gene transcription in uveal melanoma. Supplementary Figure S7. EZH2 mediates motility of UM cells via RhoGDIγ-Rac1 axis. Supplemetary Figure S8. EZH2 facilities liver metastasis of UM in NOG mice. Supplementary Figure S9. The expression of BAP1 and EZH2 is parallel in UM cells. [file 12943_2020_1173_MOESM2_ESM.pdf]

Supplementary Figure S1

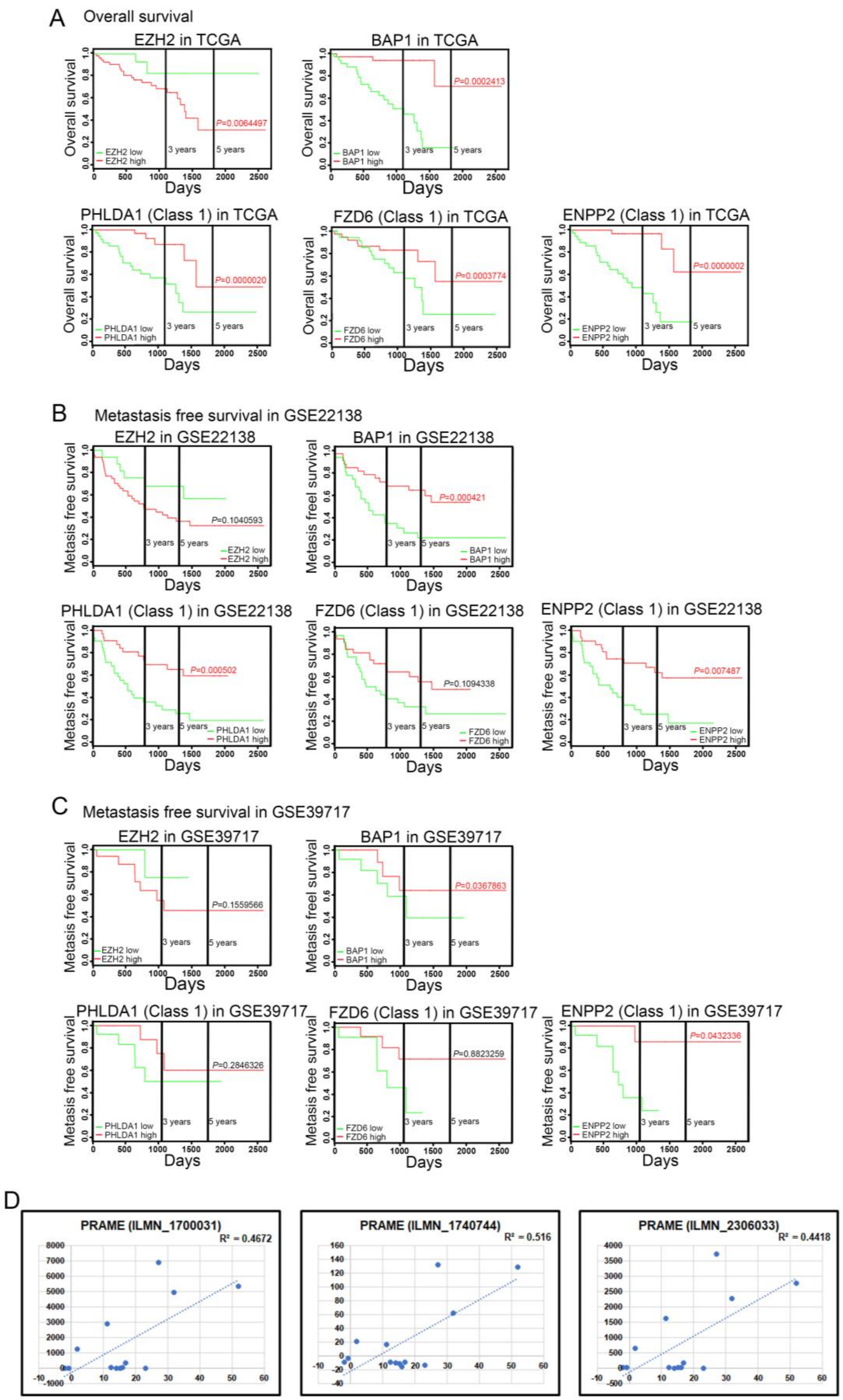

Supplementary Figure S1.

The prognostic factors of EZH2, BAP1, and Class 1 vs 2 GEP in the correlation of UM overall survival and metastasis-free survival in published database. A, The correlation of EZH2, BAP1 and the optimal three-gene sets (*PHLDA1, FZD6, and ENPP2*) which accurately predict class label (Class 1 vs 2 GEP) in UM patients overall survival in TCGA database. B-C, The correlation of EZH2, BAP1 and the optimal three-gene sets (*PHLDA1, FZD6, and ENPP2*) which accurately predict class label (Class 1 vs 2 GEP) in UM patients metastasis-free survival in GSE22138 and GSE39717 database. D, The correlation of EZH2 and PRAME in a cohort of 13 UM patient (GSE73625). ILMN numbers are unique identifier for the probes of PRAME.

Supplementary Figure S2

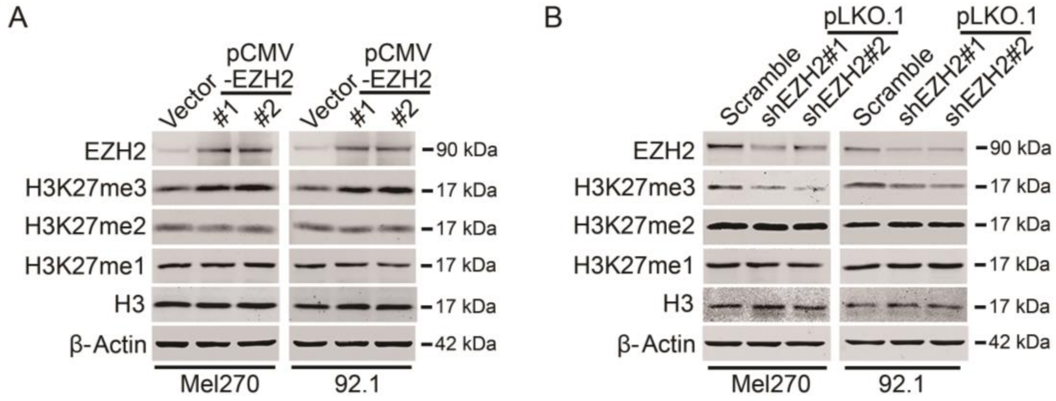

Supplementary Figure S2. Verification of EZH2 overexpression and knockdown in UM cells. Mel270 and 92.1 cells were transfected with plasmids encoding vector or pCMV-EZH2 (two different clones) (A), or infected with lentivirus particles containing scramble or two fragments shRNAs targeting EZH2 (B). Cell lysates were subjected to Western blotting analysis.

Supplementary Figure S3

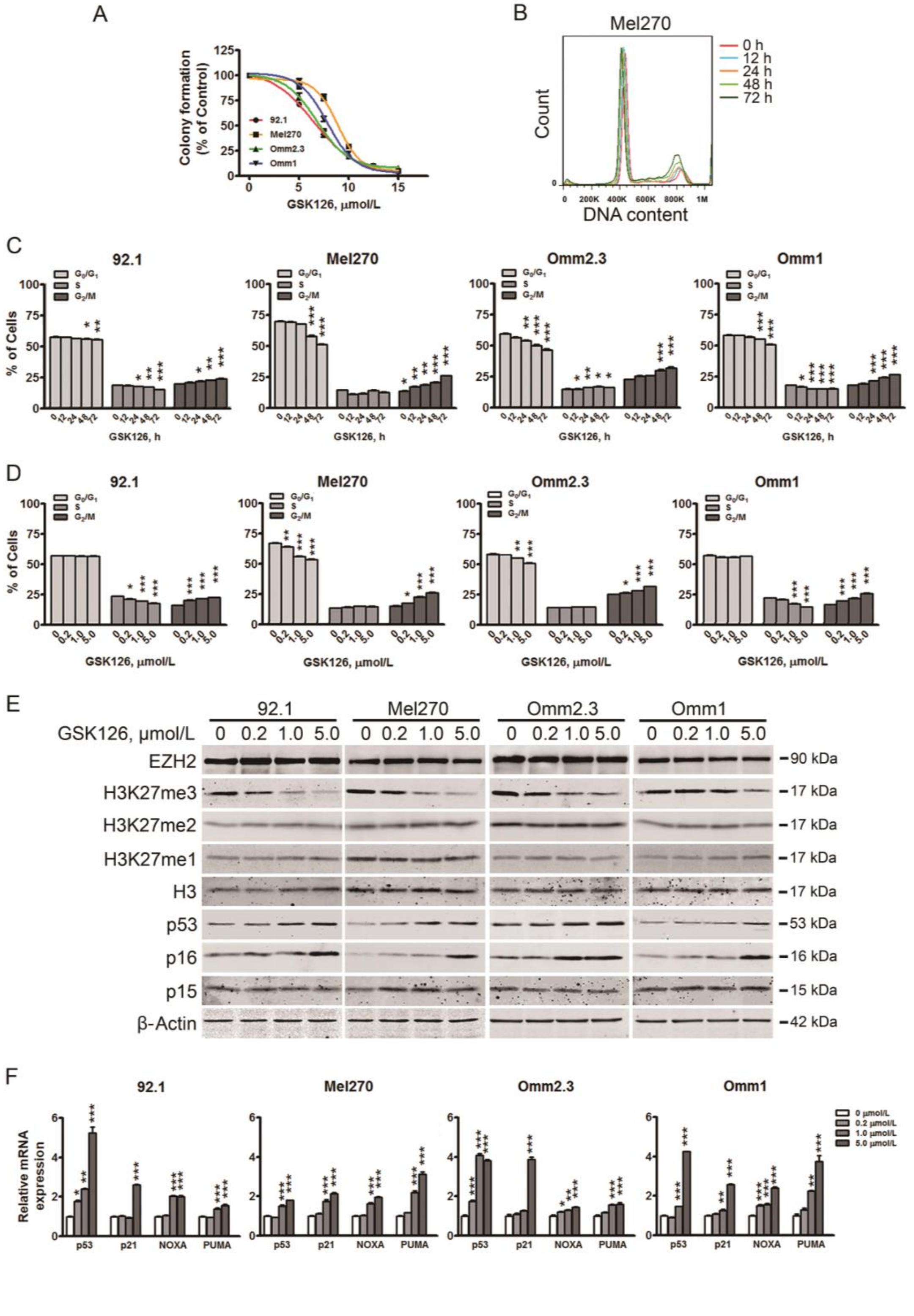

Supplementary Figure S3.

Pharmacologic inhibition of EZH2 induces G_2/M phase arrest and activation of p53. A, GSK126 diminished the clonogenicity of UM cells. UM cells were initially incubated with escalating concentrations of GSK126 for 24 h, followed by culture in drug-free agarose for 2 weeks. B-D, Pharmacologic inhibition of EZH2 results in G_2/M phase arrest in UM cells. After incubated with GSK126 as indicated, UM cells were labeled with PI for cell cycle analysis by flow cytometry. A set of representative data of cell cycle (B) and bar charts (C and D) from 3 independent experiments were shown. *, $P<0.05$; **, $P<0.01$; ***, $P<0.0001$, one-way ANOVA, *post hoc* intergroup comparisons. E-F, Inhibition of EZH2 by GSK126 activated p53 in UM cells. UM cells were incubated with increasing concentrations of GSK126, and then subjected to Western blotting analysis with the indicated antibodies (E), and qRT-PCR analysis for the transcription of p53 downstream genes (F). *GAPDH* was served as an internal control for qRT-PCR. Data are presented as mean ± SEM. *, $P<0.05$; **, $P<0.01$; ***, $P<0.0001$, one-way ANOVA, *post hoc* intergroup comparisons.

Supplementary Figure S4

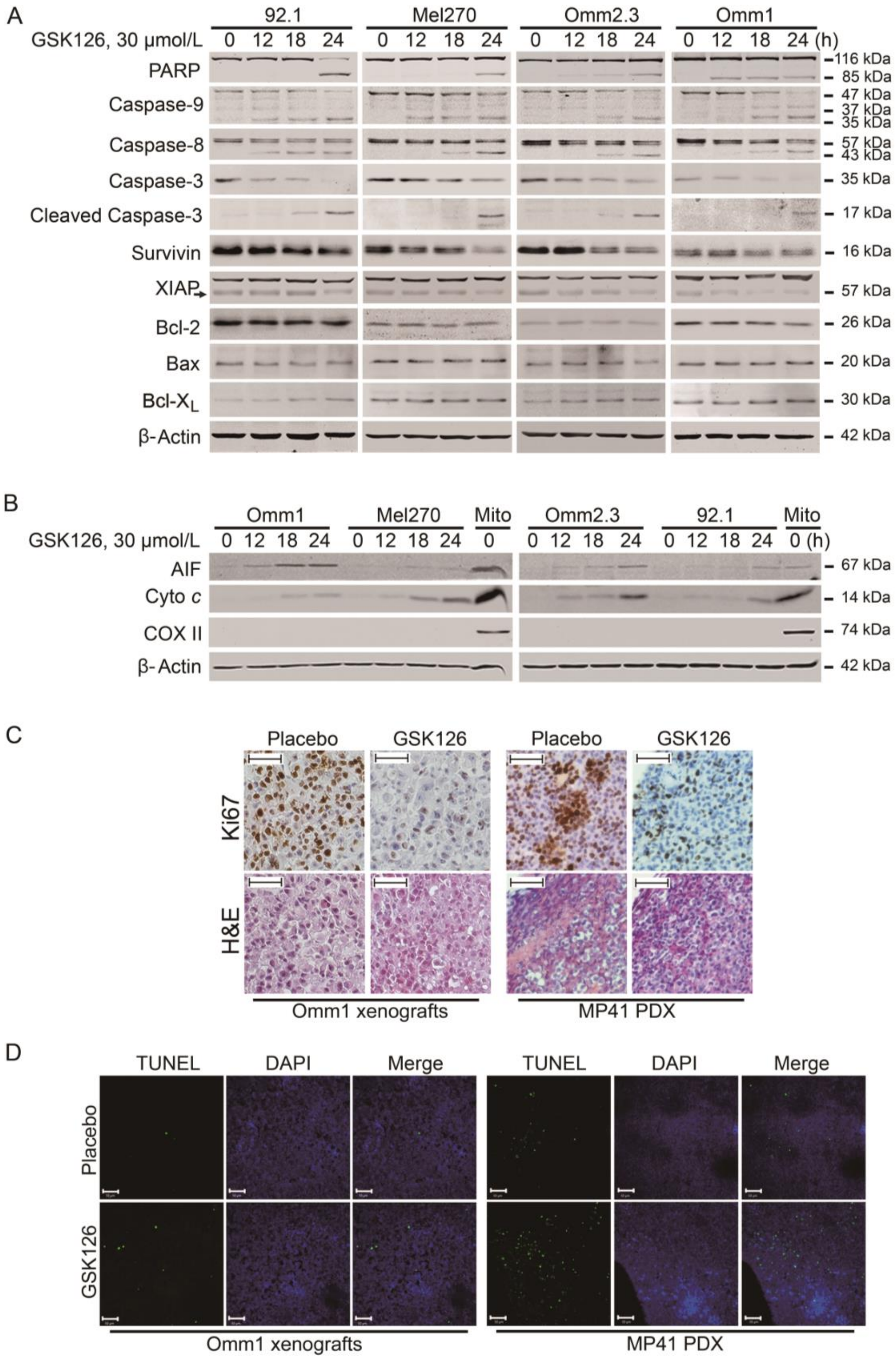

Supplementary Figure S4.

GSK126 induces apoptosis in UM via triggering intrinsic pathway. A, UM cells were treated with 30 μmol/L of GSK126 for various durations, followed by Western blotting analysis of PARP cleavage, activation of caspase family and other apoptosis-related proteins. B, GSK126 induced release of cytochrome *c* (Cyto *c*) and apoptosis-inducing factor (AIF) from mitochondrial. Cytochrome *c* oxidase subunit II (COX II) was served as a mitochondrial indicator to preclude contamination of cytosolic fractions by mitochondria (Mito). Mitochondrial lysate of Omm1 in RIPA was for the left panel, and Omm2.3 was for the right panel. C-D, GSK126 abrogated the growth and induced apoptosis in xenografted Omm1 tumor and MP41 PDX in NOD/SCID mice. IHC staining of Ki67 and H&E staining of Omm1 xenografts and MP41 PDX treated with placebo or GSK126 was applied. Representative photos were shown. Original magnification, 200× (Scale bar, 50 μm), Olympus IX71 (C). TUNEL assay of Omm1 xenografts and MP41 PDX treated with placebo or GSK126 was applied. Representative photos were shown. Original magnification, 200× (Scale bar, 50 μm), Zeiss LSM710 (D).

Supplementary Figure S5

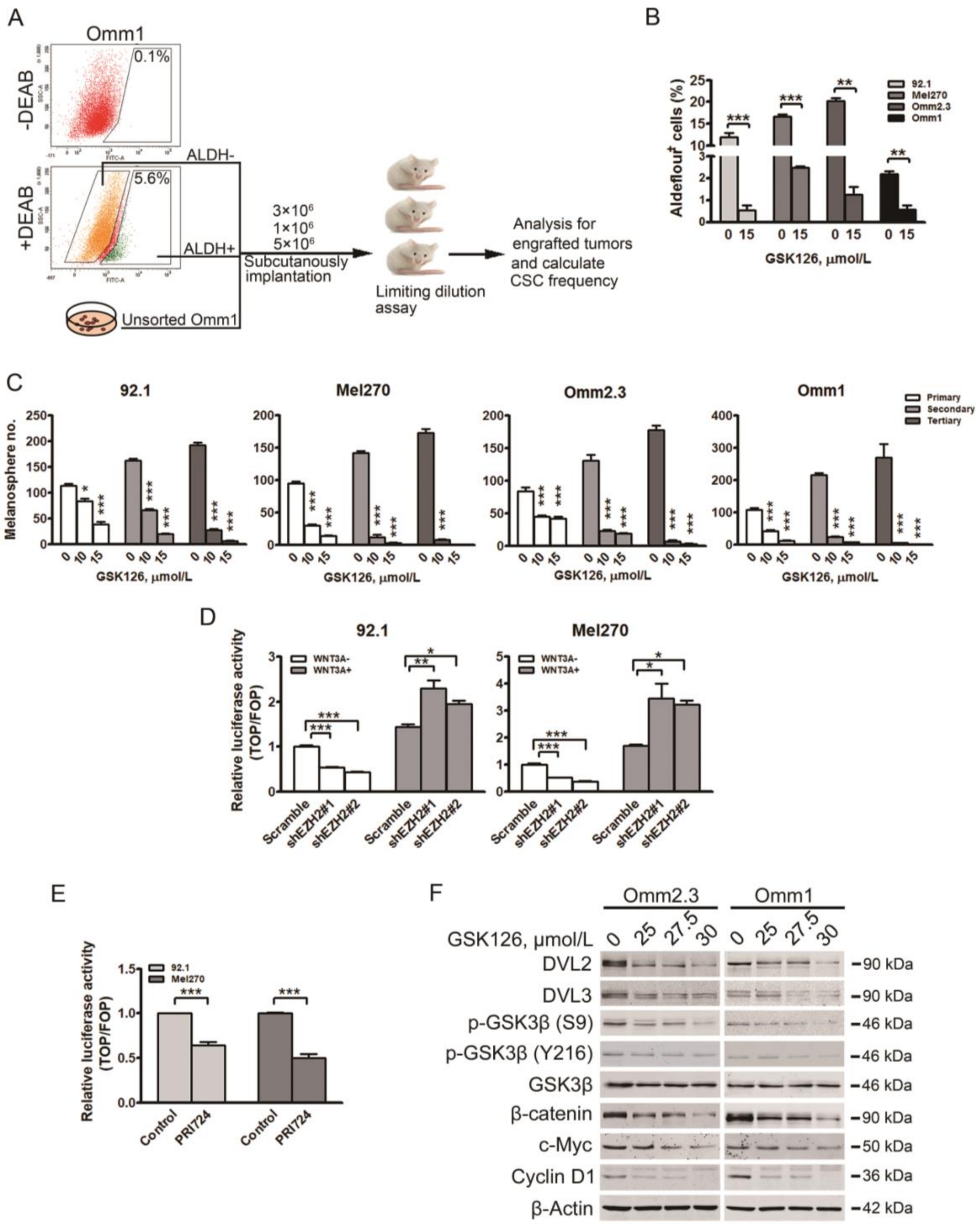

Supplementary Figure S5.

EZH2 confers maintenance of cancer stem cells (CSCs) in uveal melanoma involving Wnt/β-catenin signaling. A, Schematic of limiting dilution assay for CSC frequency of sorted ALDH+, ALDH- and unsorted Omm1 cells. B, Inhibition of EZH2 by GSK126 decreased ALDH+ cell population. UM cells were cultured with 15.0 µmol/L of GSK126 for 24 h; the ALDH+ cell population was analyzed with a flow cytometer. Bar charts from 3 independent experiments were shown. **, P<0.01; ***, P<0.0001, Student's t test. C, Inhibition of EZH2 by GSK126 decreased melanosphere formation and serially-replating ability of UM cells. *, P<0.05; **, P<0.01; ***, P<0.0001, one-way ANOVA, *post hoc* intergroup comparisons. D, 92.1 and Mel270 cells with scramble or depletion of EZH2 were transfected with TOP-flash or FOP-flash combined with *Renilla* for 24 h, followed by incubation in the absence or presence of recombinant WNT3A for another 6 h. Cell lysates were subjected to dual-luciferase reporter assay. *, P<0.05; ***, P<0.0001, one-way ANOVA, *post hoc* intergroup comparisons. E, After transfected with TOP-flash or FOP-flash combined with *Renilla* for 24 h, 92.1 and Mel270 cells were treated with β-catenin inhibitor PRI724 for 24 h, followed by dual-luciferase reporter assay. ***, P<0.0001, Student's t test. F, Omm2.3 and Omm1 cells were treated with GSK126 as indicated for 24 h, and then subjected to Western blotting analysis with the indicating antibodies.

Supplementary Figure S6

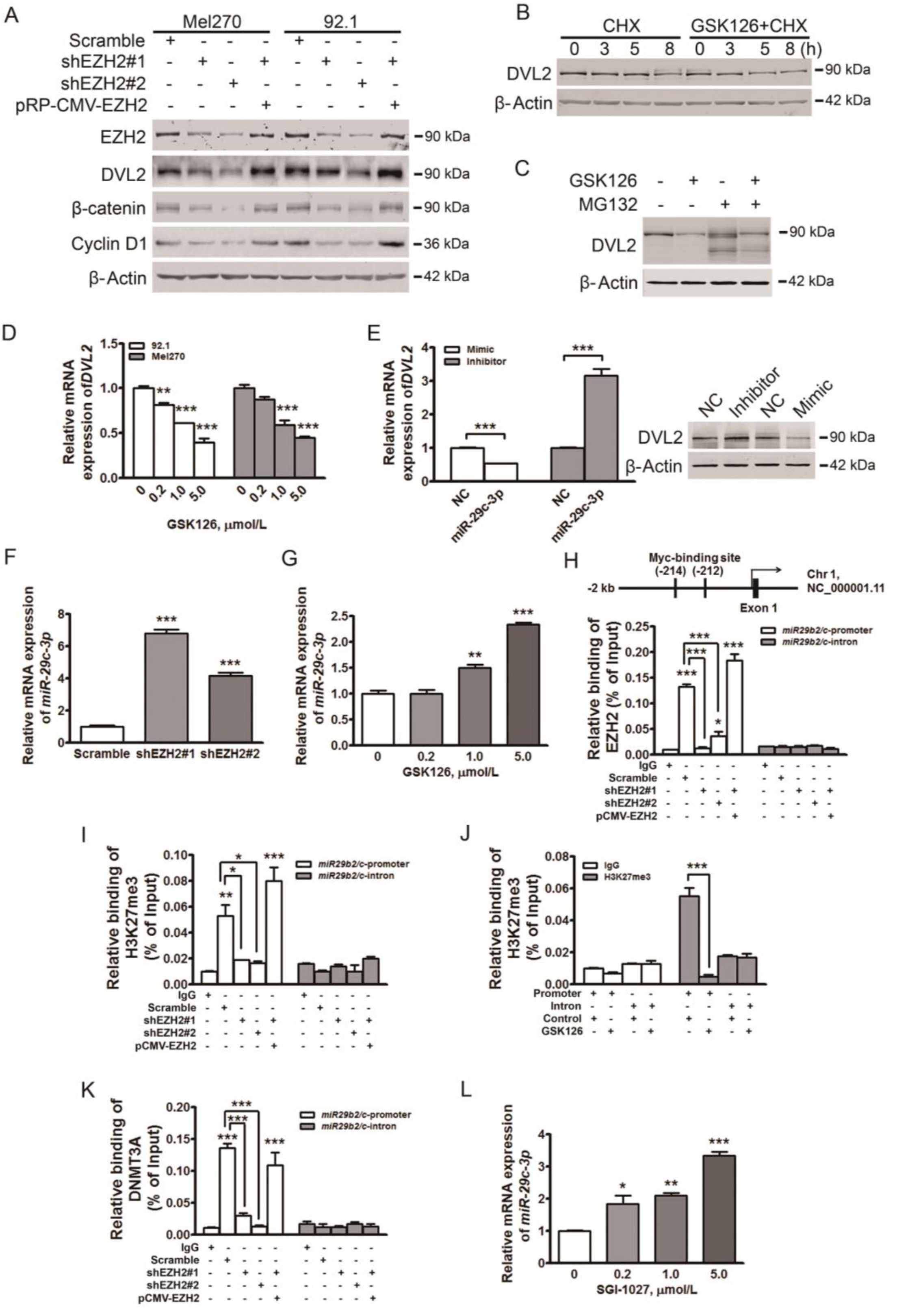

Supplementary Figure S6

EZH2 confers maintenance of cancer stem cells via suppressing *miR-29b2/c* gene transcription in uveal melanoma. A, EZH2 mediated Wnt/β-catenin signaling transduction. Mel270 and 92.1 cells transduced with lentiviral shRNA against EZH2 in the absence or presence of EZH2-encoding constructs were subjected to Western blotting analysis. B, Chase-experiments for turnover rate of DVL2 protein. Mel270 cells were treated with or without GSK126 in absence of cycloheximide (CHX) for Western blotting analysis. C, GSK126-induced DVL2 degradation was not sufficiently rescued by MG132. Mel270 cells were treated with GSK126 in absence of MG132 for Western blotting analysis. D, *DVL2* mRNA levels were declined with GSK126 treatment. **, P<0.01; ***, P<0.0001, one-way ANOVA, *post hoc* intergroup comparisons. E, miR-29c-3p mediated DVL2 expression. Mel270 cells were transfected with inhibitor, mimic of miR-29c-3p, or negative controls for qRT-PCR (*left*) and Western blotting analysis (*right*). ***, P<0.0001, Student t test. F-G, EZH2 depletion increased mature miR-29c-3p expression. Mel270 cells were treated with GSK126 for 48 h, followed by qRT-PCR assay (F). EZH2 silence in Mel270 led to elevated expression of miR-29c-3p as detected by qRT-PCR analysis (G). **, P<0.01, ***, P<0.0001, one-way ANOVA, *post hoc* intergroup comparisons. H, EZH2 was recruited to the promoter of *miR-29b2/c*. Mel270 cells were transduced with lentiviral shRNA against EZH2 in the absence or presence of EZH2-encoding constructs, followed by ChIP assay. Primers were for *miR-29b2/c* promoter region amplification covering Myc-binding sites (-214 and -212 bp away from exon1) on chromosome 1, NC_000001.11 (207802443..207802523). *, P<0.05; ***, P<0.0001, one-way ANOVA, *post hoc* intergroup comparisons. I-J, ChIP assay showed EZH2 depletion inhibited the recruitment of H3K27me3 to the promoter of *miR-29b2/c* in Mel270 cells (I). Mel270 cells were treated with GSK126 (5.0 µmol/L) for 48 h, followed by ChIP assay (J). *, P<0.05; **, P<0.01; ***, P<0.0001, one-way ANOVA, *post hoc* intergroup comparisons. K-L, EZH2 depletion in Mel270 impeded the binding of DNMT3A to *miR-29b2/c* gene promoter (K). Mel270 cells were treated with SGI-1027 for 48 h, followed by qRT-PCR assay (L). *, P<0.05; **, P<0.01; ***, P<0.0001, one-way ANOVA, *post hoc* intergroup comparisons.

Supplementary Figure S7

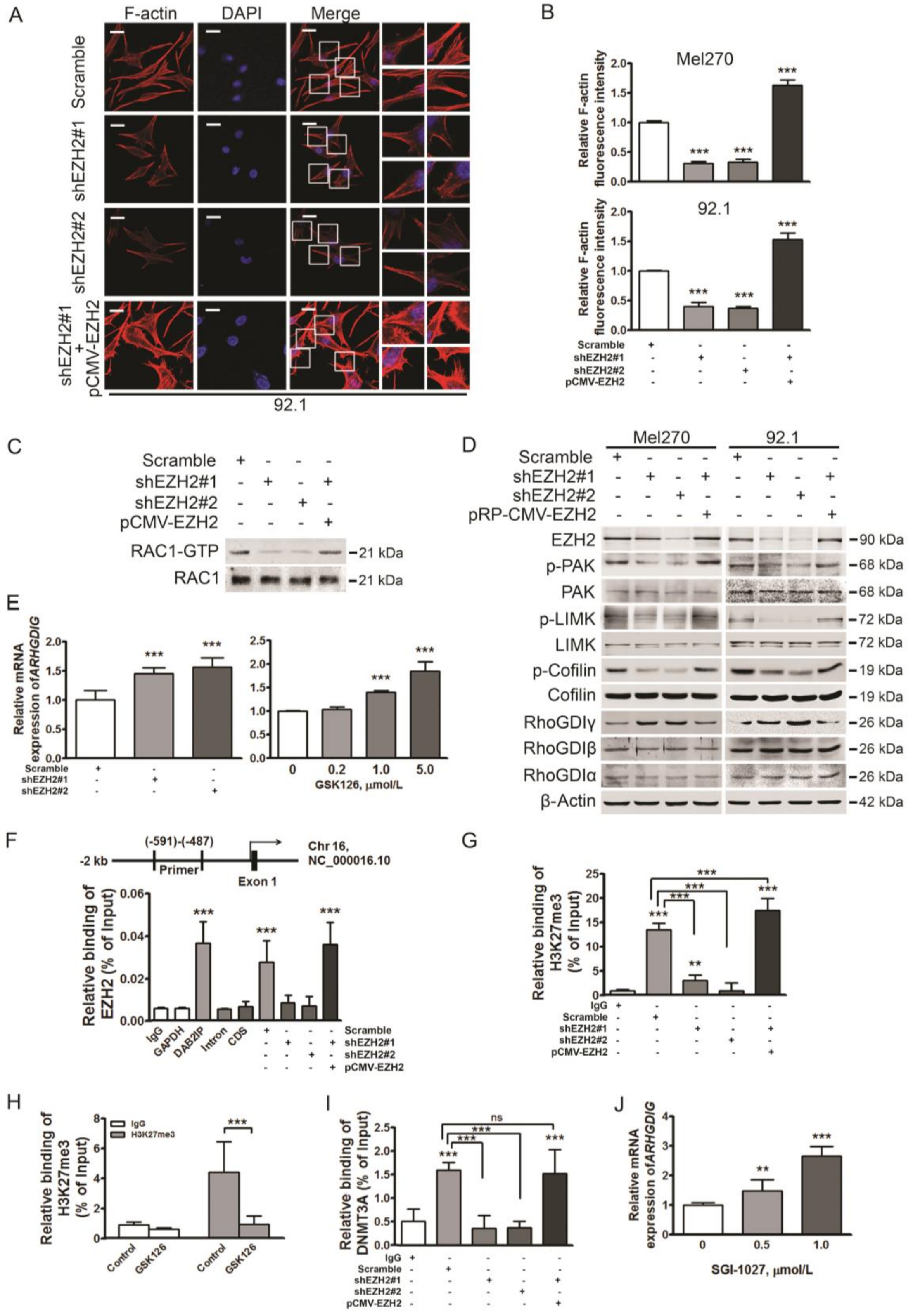

Supplementary Figure S7.

EZH2 mediates motility of UM cells via RhoGDIγ-Rac1 axis. A-B, EZH2 knockdown repressed the polymerization of F-actin. Mel270 and 92.1 cells were silenced by lentiviral shRNA against EZH2 in the absence or presence of forced re-expression by EZH2-encoding constructs, and then subjected to immunofluorescence analysis for F-actin. Zeiss LM710, oil lens, 63× (scale bar, 20 μm) (A). The fluorescence intensity of F-actin normalized relative to scramble from 3 independent fields was analyzed (B). ***, $P<0.0001$, one-way ANOVA, *post hoc* intergroup comparisons. C, EZH2 silencing inhibited the activity of Rac1. Mel270 cells were transduced with lentiviral shRNA against EZH2 in the absence or presence of EZH2-encoding constructs. Cell lysates were processed with a Rac1-GTP pull-down assay, and then subjected to Western blotting analysis with Rac1 antibody. D, EZH2 mediated PAK1-LIMK-Cofilin signaling pathway through RhoGDIγ. Mel270 and 92.1 cells were transduced with lentiviral shRNA against EZH2 in the absence or presence of EZH2-encoding constructs, and then subjected to Western blotting analysis with the indicating antibodies. E, Depletion of EZH2 upregulates the expression of *ARHGDIG*. Mel270 cells were silenced of EZH2 or treated with GSK126 followed by qRT-PCR analysis. F-H, EZH2 depletion suppressed the recruitment of EZH2 (F) and H3K27me3 (G) on the promoter of *ARHGDIG* gene. Mel270 cells transduced with lentiviral shRNA against EZH2 in the absence or presence of EZH2-encoding constructs, and ChIP assay was conducted. Primers of *ARHGDIG* were for amplification -591 - -487 bp upstream away from exon 1 on Chromosome 1, NC_000016.10 (280606..283003) as shown in the schematic. *GAPDH* served as negative control. *DAB2IP* served as positive control. Intron and CDS regions of *ARHGDIG* gene were also included. ***, $P<0.0001$, one-way ANOVA, *post hoc* intergroup comparisons. Mel270 cells were treated with GSK126, followed by ChIP assay. ***, $P<0.0001$, Student's *t* test (H). I-J, DNMT3A was recruited to the promoter of *ARHGDIG*. Mel270 cells were subjected to ChIP assay (I). Mel270 cells were treated with SGI-1027 followed by qRT-PCR analysis (J). **, $P<0.01$; ***, $P<0.0001$; one-way ANOVA, *post hoc* intergroup comparisons.

Supplementary Figure S8

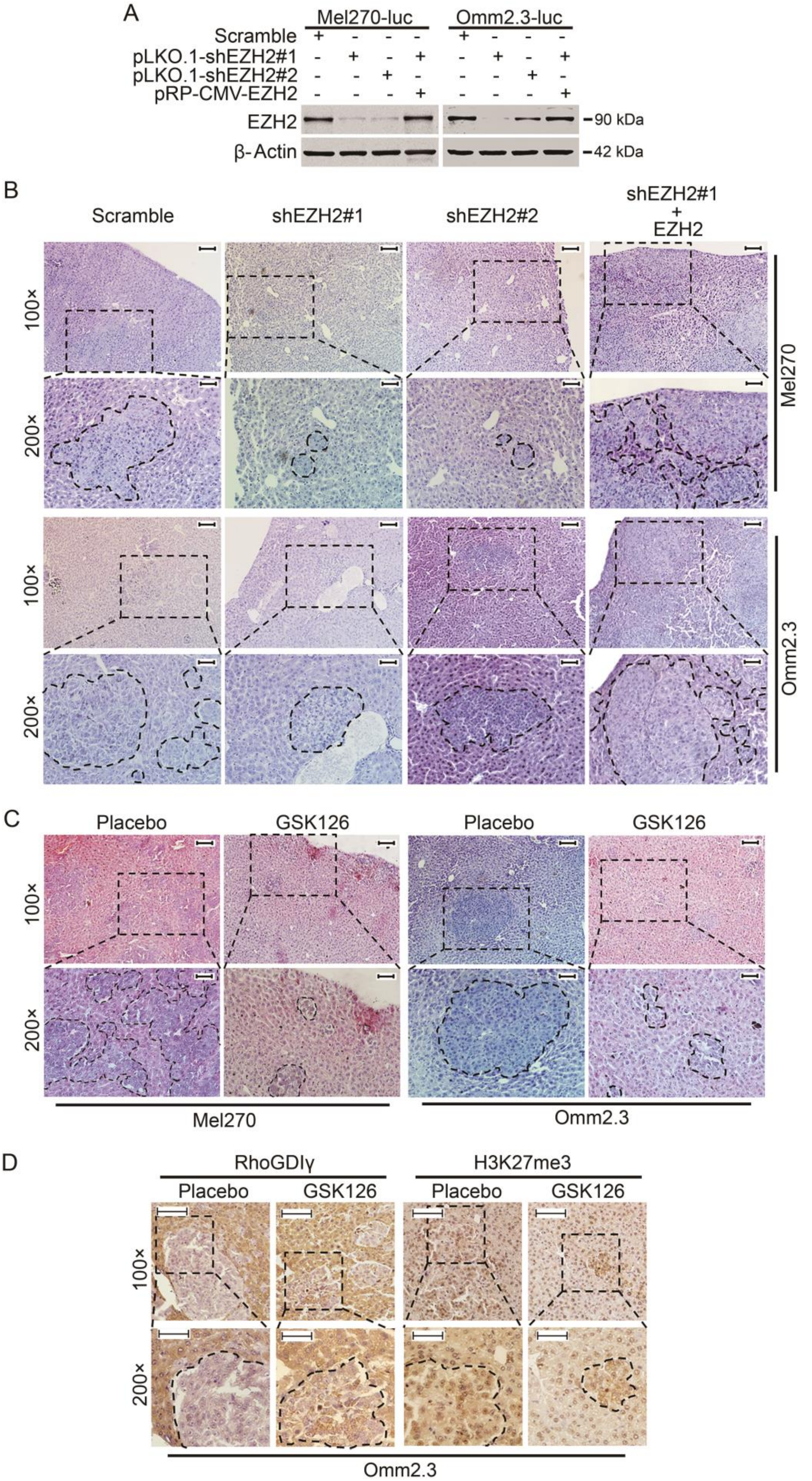

Supplementary Figure S8.

EZH2 facilitates liver metastasis of UM in NOG mice. A, Mel270-luc and Omm2.3-luc cells were stably knockdown of EZH2 by lentiviral particles carrying pLKO.1-shEZH2, with or without EZH2 rescue by transfection of plasmid encoding EZH2. Western blotting analysis of EZH2 to confirm the expression was applied. B, H&E staining of liver sections dissected from NOG mice bearing Mel270-luc or Omm2.3-luc cells with EZH2 knocked down or rescued. Photos were recorded by Olympus IX71. Original magnification was 100× (Scale bar, 100 μm) and 200× (Scale bar, 50 μm). C, H&E staining of liver sections dissected from Mel270-luc or Omm2.3-luc bearing mice treated with placebo or GSK126. Photos were recorded by Olympus IX71. Original magnification was 100× (Scale bar, 100 μm) and 200× (Scale bar, 50 μm). D, IHC staining of RhoGDIγ and H3K27me3 in metastatic liver sections from Omm2.3-luc-bearing NOG mice after treatment of placebo or GSK126. Photos were recorded by Olympus IX71. Original magnification was 100× (Scale bar, 100 μm) and 200× (Scale bar, 50 μm).

Supplementary Figure S9

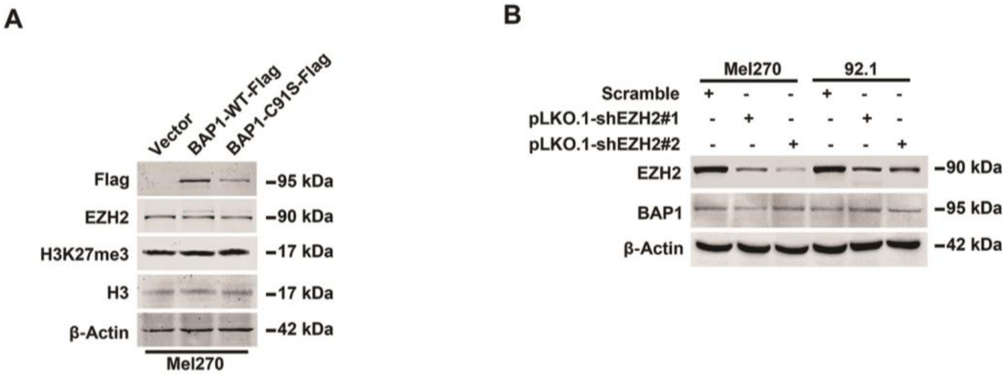

Supplementary Figure S9.

The expression of BAP1 and EZH2 is parallel in UM cells. A, Mel270 cells were transfected with plasmids encoding vector, pIRE-CMV-BAP-WT-Flag or pIRE-CMV-BAP-C91S-Flag. Cells lysates were subjected to Western blotting analysis. B, Mel270 and 92.1 cells stably knocked down of EZH2 were subjected to Western blotting analysis.
