# Supplementary material for: Verification of EZH2 as a druggable target in metastatic uveal melanoma
Source: Mol Cancer. 2020 Mar 4;19:52. doi: 10.1186/s12943-020-01173-x (PMC7055080; doi:10.1186/s12943-020-01173-x)
Supplement: Supplementary file 3 — Additional file 3. Supplementary Materials and Methods. [file 12943_2020_1173_MOESM3_ESM.doc]

**Supplementary Materials and Methods**

*Reagents and DNA constructs*

GSK126 and SGI-1027 (DNMT3A inhibitor) were purchased from Selleckchem (Shanghai, China). MG132, LiCl, cycloheximide (CHX), propidium iodide (PI), and shRNAs targeting 3’-UTR (#1) and CDS (#2) of EZH2, shRNAs targeting Survivin were from Sigma-Aldrich (Shanghai, China). SB216763 and PRI724 were from MedChemExpress (Shanghai, China). Texas Red-Phalloidin was from Molecular Probes (Eugene, OR). Plasmid pCMV-EZH2 was from Addgene (Cambridge, MA) . Plasmid pRP-CMV-EZH2 was from Cyagen (Guangzhou, China) . Plasmids encoding pTSB-vector and pTSB-Survivin were from Transheep (Shanghai, China) Mimics and inhibitors: mirVana® miRNA inhibitor miR-29c-3p (4464084) and negative control (4464076); mirVana® miRNA mimic miR-29c-3p (4464066), and negative control (4464058), were from ThermoFisher (Shanghai, China).

*Antibodies*

Antibodies against poly (ADP-ribose) polymerase (PARP), Bcl-2, XIAP, cytochrome *c*, phospho-GSK3β (Y216), p16, β-catenin and c-Myc, were from BD Biosciences (San Jose, CA); phospho-PAK1 (T423)/PAK2 (T402), PAK1, phospho-cofilin (S3), cofilin, MMP2, phospho-GSK3β (S9), caspase-3, -8, -9, cleaved caspase-3, DVL2, Axin2 and active Rac1 detection kit were from Cell Signaling Technology (Beverly, MA); apoptosis-inducing factor (AIF), EZH2, phospho-LIMK1/2 (Y507/T508), LIMK, MMP9, p57, H3K27me3, H3K27me1, H3K27me2 were from Millipore (Billerica, MA); E2F1, HA-probe, Bax, Bcl-X_L_, p53, p15, H3, RhoGDIα, RhoGDIβ, RhoGDIγ, GSK3β, Cyclin D1, DVL3, proliferating cell nuclear antigen (PCNA) and DNMT3A were from Santa Cruz Biotechnology (Santa Cruz, CA); anti-Survivin was from Novus Biologicals (Littleton, CO); β-actin and tubulin were from Sigma-Aldrich (Shanghai, China); cytochrome *c* oxidase subunit II (COX II) was from Molecular Probes (Waltham, MA); HBM45 was from DAKO (Santa Clara, CA).

*Immunohistochemistry staining and evaluation of EZH2*

Tumor sections (4.0 μm) of paraffin embedded UM patient tissues were stained with anti-EZH2 antibody using the AEC MaxVision kit (Maixin, Fuzhou, China) after melanin bleach according to the manufacturer’s instructions. The protein levels of EZH2 were semi-quantitatively evaluated according to the percentage of positive cells: 0% (score: 0), <10% (score: 1), 10–40% (score: 2), 41–70% (score: 3), and >70% (score: 4). Intensity of the staining was scored as weak (score: 1), moderate (score: 2), and strong (score: 3). Values of the two parameters were multiplied for possible scores ranking from 0 to 12. Finally, EZH2 expression in the specimens were classified into four groups: negative (score: 0), low (scores: 1–3), moderate (scores: 4–8), and high (scores: 9–12) .

*Real-time quantitative PCR (qRT-PCR)*

Total mRNA extracted with TRIzol reagent (Invitrogen, Shanghai, China) was reverse-transcribed into cDNA with maxima first strand cDNA synthesis kit (ThermoFisher, Shanghai, China). cDNA (60 ng) was used for qRT-PCR with the SYBR Premix Ex Taq Kit (Takara, Dalian, China) on CFX96 Real-Time Detection System (Bio-Rad Laboratories, Hercules, CA) according to the manufacture’s recommended protocol. The specific primers for the amplification cDNA were listed in Supplementary Table S5.

*Establishment of stable cell lines*

The lentivirus was produced by transient transfection with the constructs [pLKO.1-puro-empty vector (scramble), pLKO.1-puro-shEZH2, or pLKO.1-puro-shSurvivin, pTSB-puro-vector or pTSB-puro-Survivin] in 293T cells. Viral supernatants were harvested at 48 and 72 h after transfection and filtered through 0.45-µm filters. Mel270, 92.1, Mel270-luc and Omm2.3-luc cells were then infected with lentiviral supernatants followed by selection with 0.5 µg/mL puromycin for 1 w.

*Western blotting analysis*

The whole cell lysates were prepared in RIPA buffer (1×PBS, 1% NP-40, 0.5% sodium deoxycholate, 0.1% SDS) containing freshly added 10 mmol/L glycerophosphate, 1 mmol/L Na_3_VO_4_, 10 µg/mL pepstatin A, 5 µg/mL aprotinin, 10 µg/mL leupeptin, 10 mmol/L NaF, and 1 mmol/L phenylmethylsulfonyl fluoride . The cytosolic fractions for detecting cytochrome *c* and AIF in the cytosol were prepared with digitonin extraction buffer (10 mmol/L PIPES pH 6.8, 0.015% digitonin, 300 mmol/L sucrose, 100 mmol/L NaCl, 3 mmol/L MgCl_2_, 5 mmol/L EDTA) as described previously . Cell lysates were subjected to SDS-PAGE and immunoblotting analysis. The membranes were incubated with IRDye 680CW or IRDye 800CW secondary antibodies (LI-COR) for 1 h at room temperature and scanned with the Odyssey infrared imaging system (LI-COR) .

*Cell growth curve*

After EZH2 was silenced, rescued or overexpressed, UM cells were seeded in 12-well plate at 10^4^/well and allowed growing for 7 days. Cell numbers in triple wells were determined with hemocytometer every day for growth curve.

*Anchorage-independent growth assay*

The anchorage-independent growth of UM cells was analyzed by agarose as described . Briefly, 400 μL/well of complete RPMI1640 medium containing 1% agarose (Invitrogen, Guangzhou, China) was initially added into 24-well plates. UM cells were resuspended in 400 μL complete RPMI1640 medium containing 0.5% agarose followed by seeding on the top layer of the agarose-containing medium. The plates were kept at 4 °C for 10 min to allow solidification, and then incubated at 37 °C in a humidified atmosphere containing 5% CO_2_ for 2 weeks. Colonies containing ≥50 cells were counted under an inverted phase-contrast microscope.

*Cell viability assay*

Cell viability was determined by MTS assay (Promega, Shanghai, China). Briefly, 100 μL culture medium containing 5,000 cells for each well was seeded in 96-well plate with serial concentrations of HMT inhibitors for 72 h. MTS solution (20 μL) was added to each well 4 h before culture termination. The absorbance was read on a plate reader at 490 nm. The drug concentration resulting in 50% growth inhibition (IC_50_) was determined.

*Flow cytometry analysis*

*Cell cycle analysis*

After pre-treated with different concentrations of GSK126 for 72 h, or 5.0 µmol/L GSK126 for various durations, UM cells were harvested, washed in PBS and fixed in 66% cold ethanol overnight. The cells were stained with PI at 50 μg/mL plus 10 mg/mL RNase in PBS solution for 30 min. DNA content was analyzed by flow cytometry.

*Annexin V-FITC/PI dual staining analysis*

Cells were cultured in the presence of GSK126 for 24 h or 30 μmol/L GSK126 at different durations, and then harvested, washed in PBS and incubated in annexin V binding buffer (BD Pharmingen, San Jose, CA) with 0.3% annexin V-FITC for 20 min. Cells were washed and resuspended in binding buffer. PI was added just before flow cytometry analysis.

*ALDH^+^ population detection*

Aldehyde dehydrogenase (ALDH) was assessed with aldehyde dehydrogenase-based cell detection kit (Stem Cell Technologies, Vancouver, BC, Canada). Briefly, 0.5 mL sample (containing 10^6^ cells) in the activated reagent was transferred to a fresh tube containing 5 μL of diethylaminobenzaldehyde (DEAB) reagent. After incubated at 37 ℃ for 60 min, samples were centrifuged, and supernatant was discarded. Cell pellets were resuspended in 0.5 mL of assay buffer for FACS LSR Fortessa flow cytometer .

*Subcutaneously xenografted experiments*

For Omm1 subcutaneous xenografted experiment: Omm1 cells (3 × 10^6^ cells in 200 μL PBS) were inoculated subcutaneously on the flanks of NOD/SCID mice. Tumors were measured every other day with calipers and calculated using the formula: *a*^2^ × *b* × 0.4, where *a* is the smallest diameter and *b* is the diameter perpendicular to *a*. When the tumors grew to ~50 mm^3^, the mice were randomly divided into two groups (n=8 per group) and treated with placebo (20% captisol, i.p.) or GSK126 (50 mg/kg/day, i.p.) for 2 weeks.

Mice in Omm1 subcutaneous xenografted and UM PDX models were euthanized, and tumor xenografts were immediately removed, photographed, weighed, fixed and stored at -80℃ . Tumor sections were subjected to H&E staining and IHC analysis for Ki67 antigen (Maxim, Fuzhou, China) .

For *in vivo* limiting dilution assay: (1) After treated with control or 15.0 µmol/L GSK126 for 48 h, viable Omm1 cells were harvested and subjected to subcutaneous inoculation at serial concentrations of cells (3×10^6^, 1×10^6^, 5×10^5^). When xenografts volumes in control mice with 3×10^6^ cells reached ～1000 mm^3^, the numbers of xenografts for each group were collected. (2) Sorted ALDH^+^, ALDH^-^ or unsorted Omm1 cells were subcutaneously inoculated at serial concentrations of cells (3×10^6^, 1×10^6^, 5×10^5^). When xenografts volumes in mice bearing ALDH^+^ Omm1 cells with 3×10^6^ cells reached ～1000 mm^3^, the numbers of xenografts for each group were collected. The frequency of CSCs in UM was analyzed by Poisson statistics and summarized in Supplementary Table S3 and S4.

Male NOD/SCID mice were 4~6-week-old and purchased from Vital River Laboratory Animal Technology Co. (Beijing, China). The body weight, feeding behavior, and motor activity of animals were monitored as indicators of general health. Mice were kept under specific pathogen-free conditions in the Sun Yat-sen University animal facility. All animal studies were conducted with the approval of the Sun Yat-sen University Institutional Animal Care and Use Committee.

*TUNEL assay*

TUNEL assay was conducted with Click-iT™ Plus TUNEL Assay for In Situ Apoptosis Detection (Invitrogen, Shanghai, China) in sections of paraffin-embedded Omm1 subcutaneous xenografts and UM PDX tumors, following the instructions of manufactures . Fragmentation of cellular DNA was shown green. DAPI was used to stain nucleus. Photos were recorded by Zeiss, LSM710.

*Melanosphere culture*

UM cells were silenced/overexpressed EZH2 or pretreated with GSK126 for 24 h. Viable cells (5, 000 cells/well) were resuspended in DMEM/F-12 medium (HyClone, containing 1× B27, 10 ng/mL bFGF and 20 ng/mL EGF) and plated into ultra-low attachment 24-well plates (Thermo Fisher Scientific Inc, Waltham, MA) . After 7-day incubation, numbers of melanospheres (cells ≥50) were counted. The cultures were then harvested, and re-plated (5,000 cells/well) for the secondary and tertiary rounds, respectively. Fresh medium was added every other day. Numbers of melanospheres were determined on day 7 after each round of re-plating .

*Dual-luciferase reporter assay*

Mel270 and 92.1 cells were cultured in 24-well plate (3×10^4^/well), followed by transfection with TOP-flash or FOP-flash with *Renilla* as a transfection control. Twenty four hours later, complete culture medium was replaced with or without WNT3A (20 ng/mL) in serum-free culture medium for another 6 h. Cell lysates were subjected to dual-luciferase reporter assay (Promega, Shanghai, China) by manufacture’s instruction .

*Wound healing assay*

After pre-treated with GSK126 (15.0 μmol/L) for 24 h, 92.1 and Mel270 cells were scratched with a sterile 200 μL pipette tip. Cells were washed in PBS and replaced with fresh RPMI1640 containing 10% FBS. The same wounded area of microscopic field was recorded using an inverted phase-contrast microscope at the indicating time points .

*Transwell migration and invasion assays*

The *in vitro* migration and invasion assays were performed as previously described . For the migration assay, 5,000 cells in 200 μL serum-free RPMI1640 medium were seeded in the upper chamber (inserts); for invasion assay, 4×10^4^ cells in 200 μL serum-free RPMI1640 medium were plated in matrigel (BD Biosciences, San Jose, CA) coated upper chambers. RPMI1640 medium plus 10% FBS in the bottom chamber was added as the chemo-attractant. Twenty-four hours later, the inserts were removed, and the cells on the bottom surface of the upper chambers were fixed with 3% paraformaldehyde, followed by staining with 0.5% violet. The cells in 3 random microscopic fields were counted and photographed using an inverted phase-contrast microscope.

*F-actin staining*

Mel270 and 92.1 cells grown on coverslips in 35-mm dishes were fixed with 4% paraformaldehyde for 20 min, and then permeabilized with 0.1% Triton X-100 in PBS for 10 min. After blocked with 1% BSA for 20 minutes, cells were incubated with Texas Red-X phalloidin (Life Technologies, Shanghai, China) diluted in 5 mL/200 mL PBS for 20 minutes followed by staining with DAPI (100 mg/mL) for 1 min. The glasses were mounted with anti-fade reagent. Immunofluorescence signals were observed under a confocal microscope with 63× oil lens (Zeiss, LSM710).

*MiR-29c-3p expression detection*

Human miR-29c-3p and internal control U6 snRNA were purchased from GeneCopoeia (Guangzhou, China). MiR-29c-3p gene was amplified by qRT-PCR with All-in-One miRNA qRT-PCR reagent according to the manufacturer’s instructions. In brief, reverse of transcription of miRNA was conducted with miRNA first-strand cDNA synthesis kit (2 µg of total RNA, 2.5 U Poly A polymerase, 1 µl RTase mix, 1× PAP/RT Buffer) in a 25 µL system. The reaction mix was incubated at 37 ℃ for 60 min followed by 5-min incubation at 85 ℃ to terminate the reaction. MiRNA cDNA was subjected to qRT-PCR with amplification protocol as follows: one cycle of 10 min initial denaturation at 95 ℃, 40 cycles of 10 sec denaturation at 95 ℃, 20 sec annealing at 60 ℃, and 10 sec extension at 72 ℃. Primers for miR-29c-3p (HmiRQP0375) and sn-RNA U6 (HmiRQP9001) were from GeneCopoeia (Guangzhou, China).

*Active Rac1 detection*

Active Rac1 was determined using active Rac1 detection kit . Briefly, UM cells silenced EZH2 with or without re-expression of EZH2, were harvested under nondenaturing conditions. Cell lysates were incubated with 20 µg GST-PAK1-PBD for 1 h at 4 ℃ with gentle rotation. Proteins in pull-down resin was washed and eluted in reducing sample buffer and then subjected to Western blotting analysis with anti-Rac1.

*Chromatin Immunoprecipitation (ChIP) assay*

Mel270 and 92.1 cells were silenced EZH2 with or without re-expression of EZH2. Omm2.3 and 92.1 cells were pre-treated with 15.0 µmol/L of GSK126 for 24 h. UM cells (1×10^7^) were collected and prepared with ChIP kit (Cat# 17-371, EMD Millipore) according to the manufacturer’s instructions . In brief, the cells were fixed with 1% formaldehyde to covalently crosslink proteins to DNA and harvested in SDS lysis buffer containing protease inhibitor cocktail. Then cross-linked DNA was sonicated into 200-600 bp followed by preclearance with 60 µL of protein G agarose for 1 h. The supernatant was then immunoprecipitated with 2.0 µg of antibodies (EZH2, H3K27me3 or DNMT3A) or normal rabbit IgG at 4℃ overnight for purification of associated DNA. Protein-DNA complexes were pelleted, washed, eluted and then reversed to free DNA with proteinase K by overnight incubation at 65℃. DNA was purified using spin columns. Purified DNA was then subjected to qRT-PCR with SYBR Premix Ex Taq kit (Takara, Dalian, China) following the manufactures’ instruction. The primers were listed in Supplementary Table S5.

**References**

1. Bracken AP, Pasini D, Capra M, Prosperini E, Colli E, Helin K. EZH2 is downstream of the pRB-E2F pathway, essential for proliferation and amplified in cancer. EMBO J. 2003;22:5323-35.

2. Zeng D, Liu M, Pan J. Blocking EZH2 methylation transferase activity by GSK126 decreases stem cell-like myeloma cells. Oncotarget. 2017;8:3396-411.

3. Brustmann H. Expression of cellular apoptosis susceptibility protein in serous ovarian carcinoma: a clinicopathologic and immunohistochemical study. Gynecologic oncology. 2004;92:268-76.

4. Jin B, Wang C, Li J, Du X, Ding K, Pan J. Anthelmintic Niclosamide Disrupts the Interplay of p65 and FOXM1/beta-catenin and Eradicates Leukemia Stem Cells in Chronic Myelogenous Leukemia. Clin Cancer Res. 2017;23:789-803.

5. Zhou J, Jin B, Jin Y, Liu Y, Pan J. The antihelminthic drug niclosamide effectively inhibits the malignant phenotypes of uveal melanoma in vitro and in vivo. Theranostics. 2017;7:1447-62.

6. Jin Y, Zhang P, Wang Y, Jin B, Zhou J, Zhang J, et al. Neddylation Blockade Diminishes Hepatic Metastasis by Dampening Cancer Stem-Like Cells and Angiogenesis in Uveal Melanoma. Clin Cancer Res. 2018;24:3741-54.

7. Baumann BC, Verginadis, II, Zeng C, Bell B, Koduri S, Vachani C, et al. Assessing the Validity of Clinician Advice That Patients Avoid Use of Topical Agents Before Daily Radiotherapy Treatments. JAMA Oncol. 2018;4:1742-8.

8. Cao J, Wang Y, Dong R, Lin G, Zhang N, Wang J, et al. Hypoxia-Induced WSB1 Promotes the Metastatic Potential of Osteosarcoma Cells. Cancer Res. 2015;75:4839-51.

9. Jin Y, Zhou J, Xu F, Jin B, Cui L, Wang Y, et al. Targeting methyltransferase PRMT5 eliminates leukemia stem cells in chronic myelogenous leukemia. J Clin Invest. 2016;126:3961-80.
